# Alternative splicing shapes transcriptome but not proteome diversity in *Physcomitrella patens*

**DOI:** 10.1038/s41598-017-02970-z

**Published:** 2017-06-02

**Authors:** Igor Fesenko, Regina Khazigaleeva, Ilya Kirov, Andrey Kniazev, Oksana Glushenko, Konstantin Babalyan, Georgij Arapidi, Tatyana Shashkova, Ivan Butenko, Victor Zgoda, Ksenia Anufrieva, Anna Seredina, Anna Filippova, Vadim Govorun

**Affiliations:** 1 Laboratory of Proteomics, Shemyakin and Ovchinnikov Institute of Bioorganic Chemistry, Russian Academy of Sciences, Moscow, Russia; 2 Laboratory of the Proteomic Analysis, Research Institute for Physico-Chemical Medicine, Moscow, Russia; 3 Institute of Biomedical Chemistry, Moscow, Russian Federation; 4 Russian State Agrarian University – Moscow Timiryazev Agricultural Academy, Moscow, Russia

## Abstract

Alternative splicing (AS) can significantly impact the transcriptome and proteome of a eukaryotic cell. Here, using transcriptome and proteome profiling data, we analyzed AS in two life forms of the model moss *Physcomitrella patens*, namely protonemata and gametophores, as well as in protoplasts. We identified 12 043 genes subject to alternative splicing and analyzed the extent to which AS contributes to proteome diversity. We could distinguish a few examples that unambiguously indicated the presence of two or more splice isoforms from the same locus at the proteomic level. Our results indicate that alternative isoforms have a small effect on proteome diversity. We also revealed that mRNAs and pre-mRNAs have thousands of complementary binding sites for long non-coding RNAs (lncRNAs) that may lead to potential interactions in transcriptome. This finding points to an additional level of gene expression and AS regulation by non-coding transcripts in *Physcomitrella patens*. Among the differentially expressed and spliced genes we found serine/arginine-rich (SR) genes, which are known to regulate AS in cells. We found that treatment with abscisic (ABA) and methyl jasmonic acids (MeJA) led to an isoform-specific response and suggested that ABA in gametophores and MeJA in protoplasts regulate AS and the transcription of SR genes.

## Introduction

Alternative splicing (AS) is an essential mechanism of post-transcriptional modification responsible for the transcriptome plasticity and proteome diversity of a cell. AS plays important roles in regulating many physiological processes in plants, such as plant growth and development, circadian clock function, flowering, and stress responses^1–4^. According to recent studies, 61% of genes are subjected to AS in *Arabidopsis thaliana*, whereas approximately 40% are in maize, and 33% are in rice^5–9^. The number of alternative isoforms is known to increase under plant stress^10–13^.

Under stress conditions, splicing of genes involved in responses to abiotic stress and RNA processing changes^13, 14^, thus supporting the idea that AS plays a regulatory role in stress responses^15^. However, the molecular mechanisms of AS regulation are poorly understood.

It is known that special regulatory proteins, such as serine/arginine-rich proteins (SR proteins) and heterogenic ribonucleoproteins (hnRNP), are involved in AS tuning. These proteins bind to specific nucleotide motifs^16, 17^. SR proteins are a family of highly conserved phosphoproteins that can bind directly to alternatively spliced introns^18–20^. Several studies have demonstrated that the genes encoding SR splicing factors tend to change their splicing profiles under stress conditions, suggesting a complex loop in splicing regulation^15, 21^. However, it is not clear whether SR proteins are subject to similar regulation in non-vascular plants.

Long non-coding RNAs are potentially important yet poorly studied AS regulators involved in many regulatory cellular processes^22–24^. They can regulate splicing in humans via lncRNA–pre-mRNA interactions^25, 26^, which may mask splice sites and prevent spliceosome assembly, or cause RNA to be edited via the adenosine-to-inosine (A-to-I) mechanism, resulting in splice recognition site modification^24^. For example, up to 50% of human genes are transcribed with their natural antisense transcripts (NATs)^27^, that might be associated with AS regulation^28^. Wang *et al*. identified 37 238 antisense transcripts in *A. thaliana*, making up approximately 70% of annotated mRNAs^29^; however, data on such interactions in plants are scarce.

Over 95% of mammalian multi-exon protein-coding genes have more than a single isoform^30, 31^; therefore, many studies have sought to identify RNA molecules generated by AS, and deduce their influence on cell proteomes^32–34^. However, knowledge on the impact of different splicing variants on plant proteomes is limited. Early comparative bioinformatics analysis has suggested a limited role for AS in plants in terms of functional expansion of the proteome^35–37^; only a small number of AS events have conserved effects at the protein sequence level in Arabidopsis and rice^37^. Identifying isoform-specific proteins and peptides is challenging because of the dynamic nature of the proteome, the often low abundance of splice variant-specific proteins and peptides, and the similarity of protein isoform sequences^38, 39^. Moreover, AS affects both the level of protein produced by a gene and the transcript stability, making it difficult to predict the coding potential of isoforms^40^. For example, in *A. thaliana*, approximately 18% genes generate isoforms that degrade via nonsense-mediated decay (NMD) caused by a premature termination codon (PTC)^41, 42^. In humans, 85% of mRNAs studied are predominantly transcribed as a single, major isoform^43^. A combination of mass spectrometry and transcription profile analysis can help elucidate the roles of AS in gene expression regulation creating proteome diversity.

The moss *Physcomitrella patens* is a common model organism in systems biology^44–46^. Moss has two life forms – filamentous protonemata representing the juvenile stage, and leafy gametophores representing the adult stage. Protonema filaments are used as a source of protoplasts, which are an ideal system for developmental studies because of their ability to regenerate intact plants at high frequency^47^. Approximately 50% of *P. patens* protonema genes are alternatively spliced, with the most frequent type of AS being intron retention^48, 49^. Stress factors and environmental conditions, such as exposure to light, both influence AS^48, 49^. However, the role of AS in the developmental transition from protonema to gametophore is poorly studied, as is its effect on reprogramming during protoplast isolation. There are also no data on the influence of AS on the moss proteome.

In this study, we used RNA-seq, mass spectrometry, Reverse Transcription-PCR (RT-PCR), and quantitative Reverse Transcription PCR (qRT-PCR) to investigate dynamics in the AS of genes in *P. patens*, and the influences of AS on the cell proteome during the transition from protonemata to gametophores or protoplasts. We found that the transcriptomes of gametophores, protonemata, and protoplasts differ considerably. During the developmental transition from protonemata to gametophores, changes in AS were associated with genes involved in carbohydrate and lipid metabolism, growth and development processes, and stress responses. In protoplasts, changes in AS were observed in genes related to transcription regulation, development, cell interactions, and cell response to biotic and abiotic stresses. Using mass spectrometry, we identified 1458 isoform-specific tryptic peptides, representing 616 alternatively spliced genes, thus confirming their translation.

To evaluate the factors regulating AS processes, we analyzed SR proteins and lncRNAs in *P. patens* protonemata, gametophores and protoplasts. We identified new SR gene isoforms at the transcriptome and proteome levels, and analyzed isoform-specific transcription under treatment with abscisic and jasmonic acids. We also investigated the lncRNA–mRNA and lncRNA–pre-mRNA interactions in gametophores, protonemata, and protoplasts using the identified non-coding transcripts in these cell types, and data from CANTATAdb^50^. Our results indicate that such interactions are important in AS regulation.

## Results

### Transcriptome Profiling of Protonema, Gametophore, and Protoplast Cells Reveals an Extended Catalog of Differentially Expressed Genes

**Figure 1:**
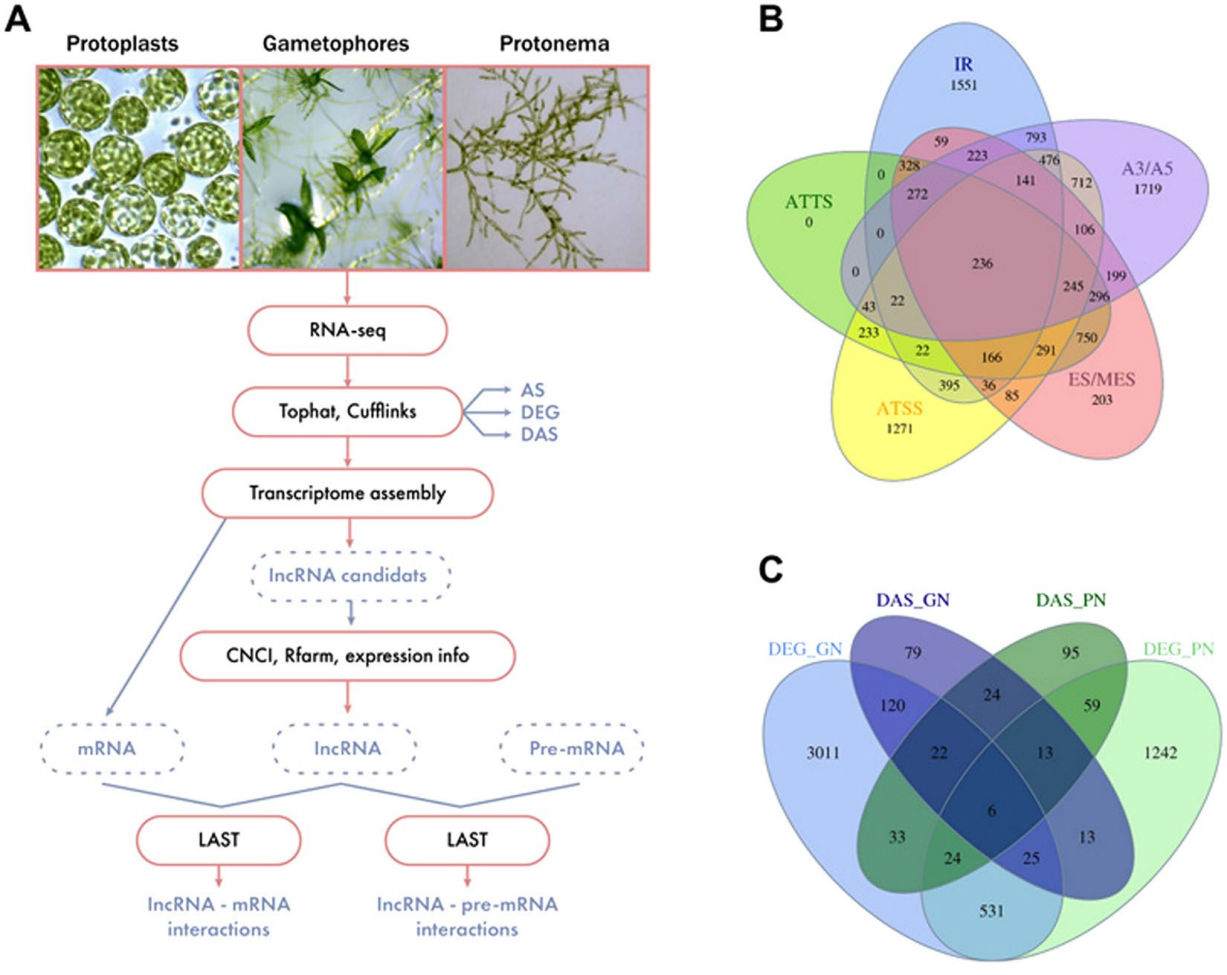
(**A**) The analysis pipeline. Transcriptome profiling data of gametophores, protonameta and protoplasts were analyzed by TopHat and Cufflinks and were searched for differentially expressed (DE) and differentially alternatively spliced (DAS) genes. LncRNAs candidates from transcriptome assembly were filtered by the Coding-Non-Coding Index (CNCI) program and BLASTed against Rfam database. The LAST alignment tool was used to calculate the potential lncRNA-mRNA and lncRNA-pre-mRNA interactions. (**B**) Venn diagram showing the different types of AS events identified in the *Physcomitrella paten*s. (**C**) Representation of differentially alternatively spliced (DAS) genes compared to differentially expressed (DE) genes in protonemata and protoplasts (DAS_PN and DEG_PN), and protonemata and gametophores (DAS_GN and DEG_GN).

To explore the transcriptomes of juvenile and mature moss life forms, and that of protoplasts, and discover which genes are differentially expressed (DE) and differentially alternatively spliced (DAS) during developmental transition or protoplastation, we used previously described data from strand-specific poly-A RNA sequenced by ABI SOLID 4.0^51^ as shown in Fig. 1A.

We discovered 23 446 transcribed genes reflecting 47 976 mRNA isoforms. Comparing gametophores and protonemata, 3772 DE genes were identified; 1906 were upregulated in gametophores and 1866 were downregulated in protonemata. Comparative analysis of protonema and protoplast transcriptomes revealed 1913 DE genes, with 1470 of these upregulated in protoplasts. We compared the transcription level of DE genes in protonemata, gametophores, and protoplasts using k-medians clustering (Supplementary Figure S1; Supplementary Table S1). In gametophores, genes involved in morphogenesis, growth, gametophore development, sexual reproduction, polysaccharide synthesis, and stress response were upregulated (see Supplementary Data S1). In protonemata, transcript accumulation was increased in genes involved in cell wall synthesis, photosynthetic processes, carbohydrate biosynthesis, cellular morphogenesis, cell size regulation, cell generation, cell cycle processes, and cytokinesis (Supplementary Figure S1; Supplementary Table S1).

### Identification of Alternatively Spliced Genes

The most abundant types of AS in *P. patens* are alternate donor and/or acceptor site and intron retention; the latter accounts for 40% of splicing in moss^48, 49^. According to our RNA-seq data, 12 043 genes had more than one isoform, suggesting that AS had taken place; 4720 of these demonstrated intron retention splicing (Fig. 1B). Of all AS variants revealed, 57.06% were previously annotated and 42.94% identified as new splice variants. Genes with isoform transcription levels that changed by more than 20% (dIF >20, see Materials and Methods for details) were selected as DAS genes for further definition to the level of transcript accumulation between different cell types. In total, we identified 514 DAS genes; 302 and 276 of these were differently spliced among protonemata and gametophores, and protonemata and protoplasts, respectively (Supplementary Table S2). DAS and DE genes were found to overlap by 4,2% and by 4,6% among protonemata and gametophores, and protonemata and protoplasts, respectively, (Fig. 1C).

There are two types of spliced introns in higher plants: U2-dependent (U2) and U12-dependent (U12) introns^52, 53^. We calculated that U2-type spliceosomes recognized 99% of AS events (120 181), and U12-type recognized only 1% (1213) in *P. patens*.

Many eukaryotic genes are alternatively spliced at their 5′-untranscribed region (UTR). In UTRs, AS modulates mRNA stability and thus overall protein expression^54, 55^. We evaluated the effect of transcripts’ 5′-UTR sequence length on the level of expression of corresponding *P. patens* gene transcripts. Isoforms with long UTRs were found to have lower expression levels (Supplementary Figure S2).

### Changes in Alternative Splicing During Transition from Juvenile to Mature Stages

**Figure 2:**
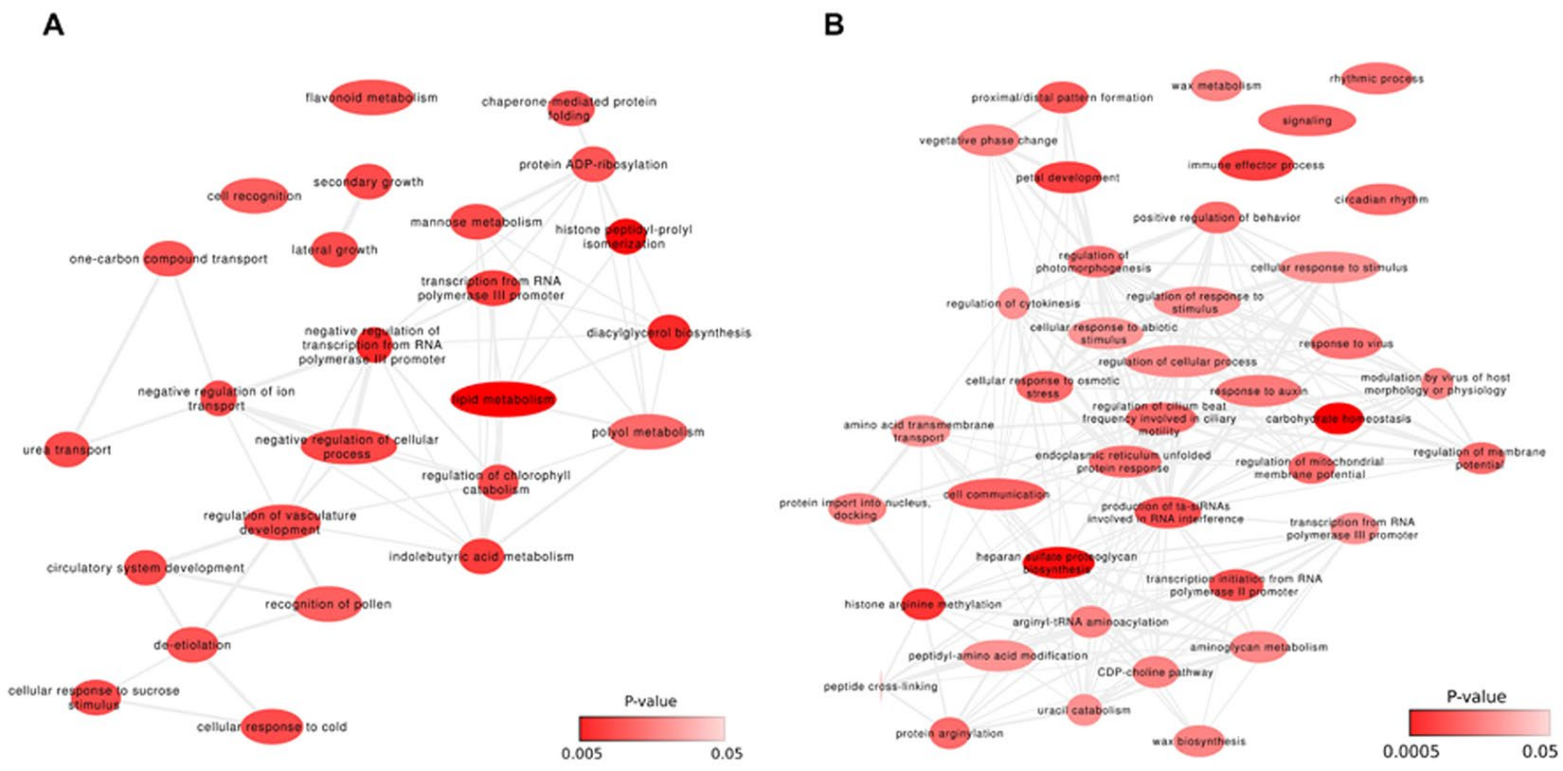
(**A**) The most frequently represented gene ontology (GO) terms of differentially alternatively spliced (DAS) genes in protonemata and gametophores. (**B**) The most frequently represented GO terms of DAS genes in protonemata and protoplasts.

Three-hundred-and-two genes were found to be DAS between the juvenile life form of the moss and the mature form (Fig. 1C). Functional analysis of these DAS genes revealed that the most frequently represented GO terms were associated with lipid metabolism (GO:0006629), carbohydrate metabolism (GO:0005975), growth and development processes (GO:0080190, lateral growth; GO:0080117, secondary growth), and response to stress conditions (GO:0071329, cellular response to sucrose stimulus; GO:0070417, cellular response to cold) (Fig. 2A; Supplementary Table S2). Examples of DAS genes were Pp1s15_135V6 (ABCG40), Pp1s324_39V6 (poly-(ADP-ribose) polymerase), Pp1s346_35V6 (vacuolar membrane H^+^-pyrophosphatase), and Pp1s309_69V6 LEA homolog (late embryogenesis abundant domain-containing protein), which are all involved in cellular response to desiccation. These results are in accordance with the specific growth conditions of the gametophore stage. Other DAS genes identified included transcription factors belonging to the AP2/ERF and NAC families (e.g., Pp1s143_82V6 and Pp1s164_37V6), PpABI3A (Pp1s143_82V6), an ABI3 transcription factor homolog, and factors regulating AS such as the SR splicing factor RSP41 (Pp1s144_89V6).

### Changes in Alternative Splicing in Response to Protoplastation

Comparing protonemata with protoplasts allowed us to identify 276 DAS genes (Fig. 1C, Supplementary Table S2). Analysis of the GO terms of these genes revealed their involvement in transcription regulation (RNA interference), development (GO:0048441; petal development), cell interactions (GO:0007154; cell communication), and response to biotic (GO:0009615; response to virus) and abiotic (GO:0009628; response to abiotic stimulus) stresses (Fig. 2B).

The DAS genes identified encode proteins with various functions, e.g. a protein kinase superfamily protein (Pp1s22_429V6), translation initiation factor IF2/IF5 (Pp1s626_4V6), transcription initiation factor TFIID subunit 7 (Pp1s111_159V6), regulatory components of ABA receptor 3 (Pp1s97_204V6.2), ARM repeat superfamily protein (Pp1s21_271V6), AP2 ERF domain-containing transcription factor (Pp1s225_102V6), pre-mRNA splicing factor SF2 **(**Pp1s28_193V6**)**, and SR splicing protein (Pp1s9_310V6).

In protoplasts, we identified 14 genes that had several isoforms with dIF >20 and a considerably increased expression (Supplementary Table S2). For example, Pp1s252_67V6 (A9TKP2) is a putative ATP-binding cassette transporter; its *A. thaliana* homolog (AT3G28860) is involved in auxin polar transport. We identified four isoforms of this gene; the transcription levels of these isoforms differed considerably between protoplasts and protonemata.

### The effect of AS on the Cell Proteome is Small

**Figure 3:**
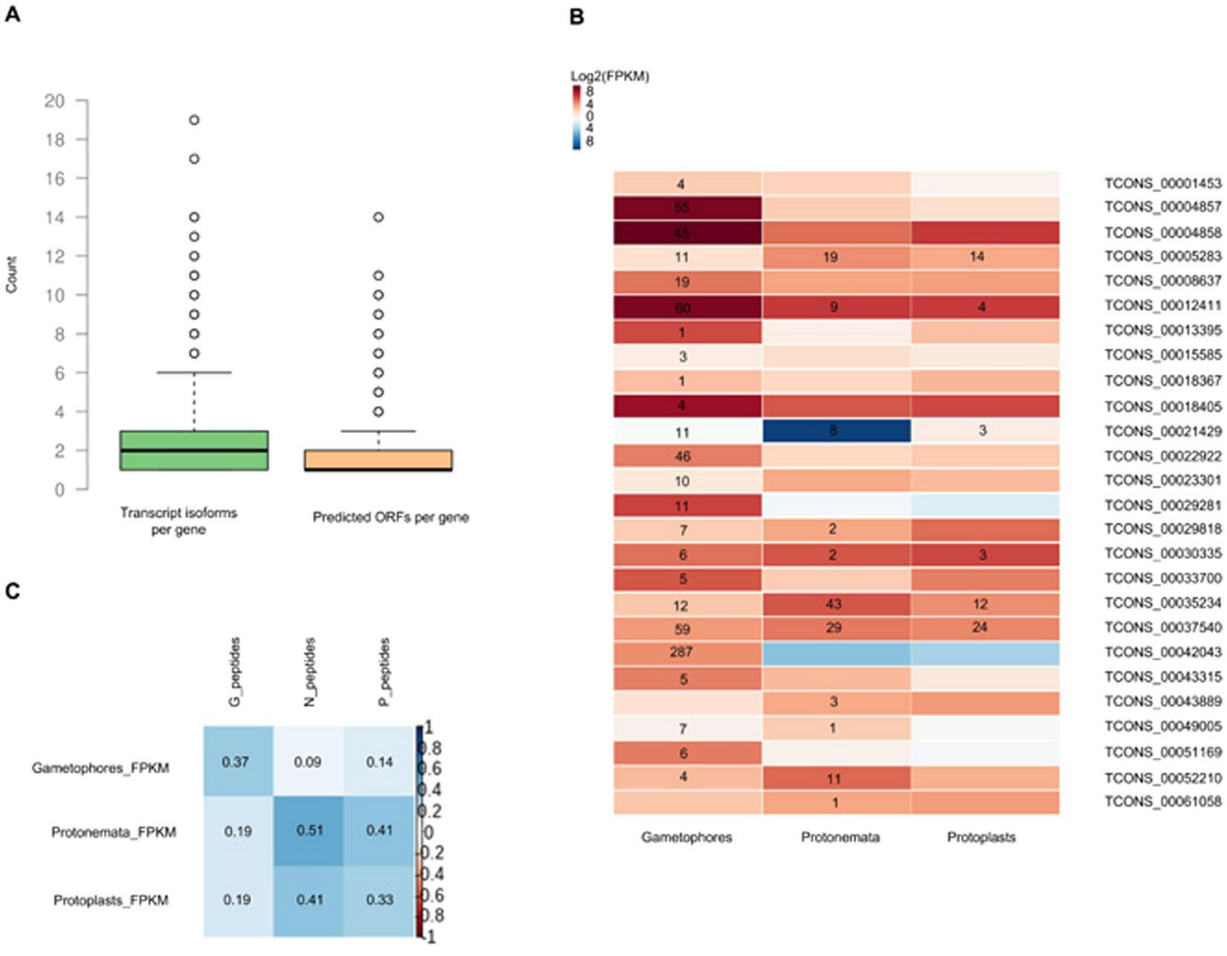
(**A**) Bar plot showing the distribution of the number of transcript isoforms and predicted ORFs per gene. (**B**) Heatmap representing the levels of isoform transcripts of 25 differentially alternatively spliced (DAS) genes. Digits indicate the number of spectra from isoform-specific peptides. (**C**) Heatmap reports the correlation between the level of transcription of alternative isoforms and the normalized values of spectra from isoform-specific peptides for gametophores (Gametophores_FPKM-G_peptides), protonemata (Protonemata_FPKM-N_peptides) and protoplasts (Protoplasts_FPKM-P_peptides); digits indicate Spearman correlation coefficients.

To estimate the effect of AS on the cell proteome, we predicted 65 846 open reading frames (ORFs) for 58 769 transcripts of 36 233 genes using TransDecoder (https://transdecoder.github.io; see Materials and Methods). We observed that the number of isoforms per gene at transcript level was larger than the number of predicted unique ORFs, thus indicating the greater diversity of mRNAs (Fig. 3A).

We created a custom database of the amino acid sequences corresponding to predicted ORFs and performed mass spectrometry to find proteins encoded by alternatively spliced isoforms. In total, 36 995 unique tryptic peptides from 17 123 predicted ORFs were identified and 7740 translated genes were confirmed. According to our data, 4597 of these genes were alternatively spliced. Overall, we identified 1458 isoform-specific peptides (ISPs) representing 616 (625 ORFs) alternatively spliced genes (Supplementary Table S3). Our approach also identified 13 genes that were not annotated in genome version 1.6. Among these, we identified 85 ISPs from 25 DAS genes (Fig. 3B); however, not all possible isoform-specific proteins could be distinguished from the same locus at the proteome level. This low number of ISP can be explained by following reasons: 1) single isoform of the gene is translated at current time and place; 2) proteins translated from different isoforms of one gene are almost identical, such that the splicing events might result in the addition of extra exons (protein tuning) by alternative transcription initiation (ATI) and alternative transcription termination (ATT) mechanisms, but not in changes to the protein-coding sequences (protein editing). For example, according to our data, the gene encoding gibberellin 3-beta-dioxygenase (Pp1s277_78V6), which is involved in the transformation of non-active gibberellin into the bioactive form, is highly expressed in protoplasts. The Pp1s277_78V6.1 isoform encodes a protein that is 79 amino acids smaller than the Pp1s277_78V6.2 isoform. We identified 16 tryptic peptides, indicating that both isoforms may be translated. However, the unique tryptic peptides, confirming the translation of the Pp1s277_78V6.2 (TCONS_00029818) isoform, were identified only in gametophore and protonema samples (Fig. 3B).

We conducted a search of AS genes for peptides that unambiguously indicated the presence of two or more splice isoforms from the same locus. However, we could only distinguish a few examples, such as Pp1s475_21V6 (amidohydrolase 2) (Supplementary Figure S3; Supplementary Table S3).

In order to exclude poor protein sequence coverage as the main reason for low rates of alternative isoforms detection, we performed *in silico* AS detection experiment to estimate the number of AS genes we would have expected to identify from our proteomic data (see details in Material and Methods). The test was repeated 100 times and the average number of AS genes from this *in silico* analysis was 66 times greater than observed in our proteomic experiments (Supplemental Figure S4). This result clearly indicates that the low number of confirmed AS isoforms at proteome level is difficult to explain away as a purely limitation of the proteomics technologies. We also found a low correlation between the level of transcription of alternative isoforms and the number of spectra from isoform-specific peptides (Fig. 3C). Thus, these results showed little correspondence between the predicted proteome plasticity shaped by AS, and observed translation of particular isoforms at the proteome level.

### The Possible Role of lncRNAs in the Regulation of Alternative Splicing

Long non-coding RNAs (lncRNAs), which comprise antisense, intergenic, and intron transcripts over 200 nucleotides long, are essential components of eukaryotic transcriptomes^56^. Interactions of lncRNAs with genomic loci, pre-mRNAs and mRNAs, play an important role in the regulation of gene expression, AS, and translation in mammals. To shed light on the influence of lncRNA on AS we analyzed the potential lncRNA–mRNA and lncRNA–pre-mRNA interactions in the transcriptomes of the three moss cell types.

**Figure 4:**
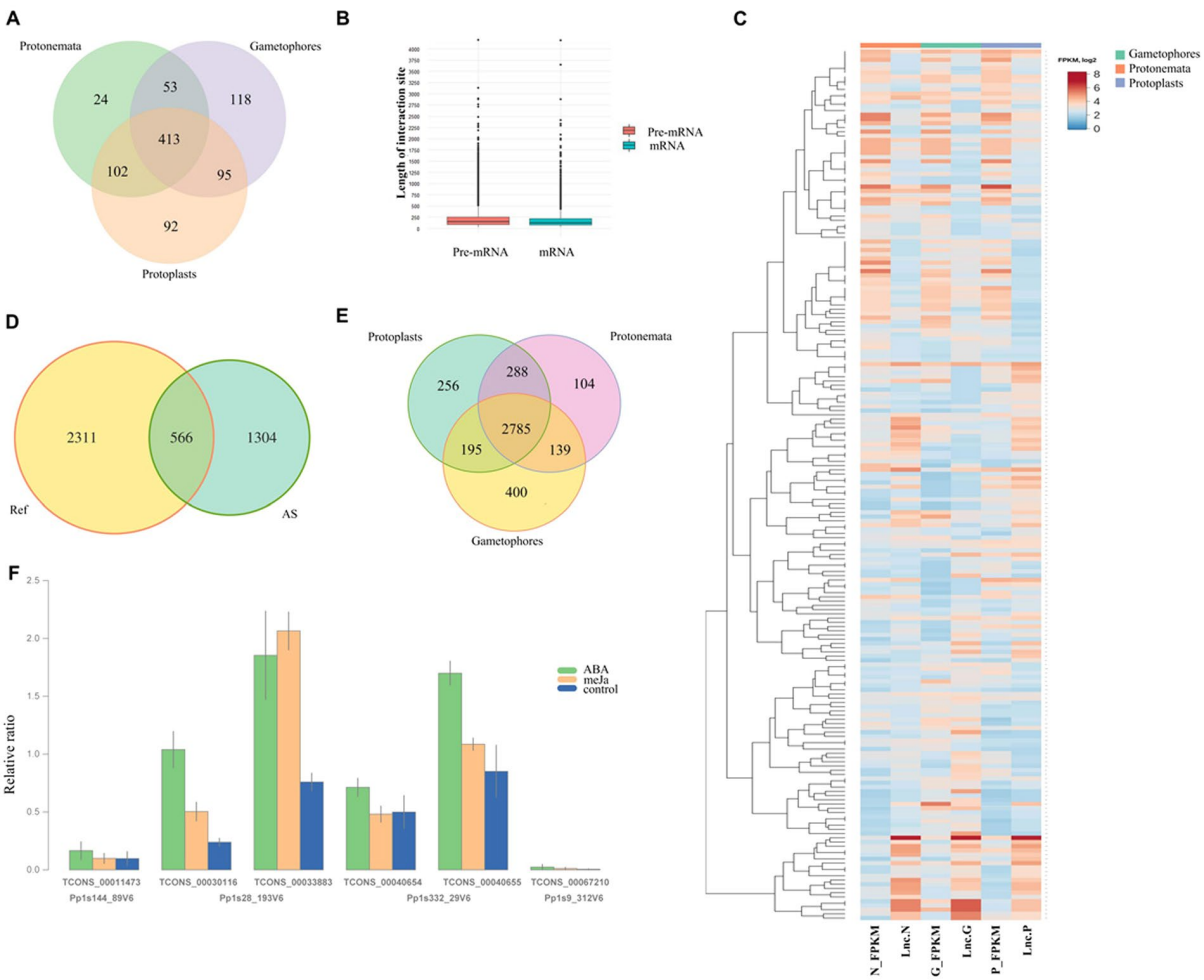
(**A**) Venn diagram showing lncRNAs distribution in gametophores, protonemata and protoplasts. (**B**) Bar plot showing the length of interaction sites between lncRNA-mRNAs and lncRNA-pre-mRNAs. (**C**) Heatmap representing expression patterns of the potentially interacted lncRNAs and mRNAs in gametophores, protonemata and protoplasts. N_FPKM, G_FPKM, P_FPKM ≥indicating the expression profiles of mRNAs in protonemata, gametophores and protoplasts, respectively. Lnc.N, lnc.G and lnc.P indicating the expression profiles of lncRNAs in protonemata, gametophores and protoplasts, respectively; (**D**) Venn diagram representing the overlap of unique interaction sites between reference (Ref) and alternative (AS) isoforms. (**E**) Venn diagram showing the landscape of lncRNA:mRNA interaction sites in gametophores, protonemata and protoplasts. (**F**) Results of quantitative polymerase chain reaction (qRT-PCR) data for serine/arginine-rich (SR) genes after treatment of protonemata with 50 µM abscisic acid (ABA) and 400 µM methyl jasmonate (MeJA). Protonemata treated with ultrapure Milli-Q water were used as a control.

Firstly, we predicted lncRNAs in gametophore, protonema, and protoplast cells using the strand-specific transcription data obtained previously^51^ (Fig. 1A). Eight-hundred-and-ninety-seven lncRNAs were found: 679 in gametophores, 592 in protonemata, and 702 in protoplasts (Fig. 4A; Supplementary Table S5). We arbitrarily chose eight identified lncRNAs to validate our RNA-seq data using RT-PCR and qRT-PCR (Supplementary Figure S5, Supplementary Table S4).

From our data set, 897 lncRNAs were used, and 2711 lncRNAs from CANTATAdb^50^; 145 (15.5%) lncRNAs overlapped the two databases. The analysis revealed 6753 interactions between lncRNAs and mRNA transcripts. Two-hundred-and-ninety-five potential RNA–RNA interactions were found between the 116 lncRNAs from our data set and 159 mRNA transcripts for 109 genes (Supplementary Table S4). The length of the interaction sites ranges from 25 to 4198 bp (Fig. 4B). A highly positive Spearman correlation (r > 0.8, *P*-value < 0.05) was found between the transcription level of mRNA and the associated lncRNAs for 57 genes suggesting possible co-regulation among them (Fig. 4C, Supplementary Table S4).

We compared the patterns of lncRNA-mRNA interactions for reference transcripts (according to moss genome annotation v1.6) vs. alternative isoforms and found that only 13.5% of interaction sites are common between the two groups of transcripts (Fig. 4D). These data emphasize AS potential to influence RNA-RNA interaction network in the transcriptomes. In addition, our results demonstrated that some of the lncRNA-mRNA interactions are tissue-specific (Fig. 4E).

To evaluate the effect of lncRNAs on AS in moss genes, we analyzed the interactions between lncRNA and pre-mRNA. We detected 5208 potential lncRNA-pre-mRNA interactions between 1221 lncRNAs and pre-mRNA for 1761 genes (Supplementary Table S4). We classified all pre-mRNA sites involved in the establishment of lncRNA-pre-mRNA interactions according to their location on pre-mRNA and found that 14% (720) of interactions occur in potential AS regions. 80% (576) of interactions were identified in the exon–intron junctions implying these sites as the most preferable for lncRNA-pre-mRNA interaction. We also found a significant overrepresentation of alternative splice junctions (Fisher’s exact test, p-value < 2.2e-16) among splice junctions interacted with lncRNAs, suggesting the importance of such interactions in the regulation of AS.

### Alterations in Transcription and Splicing of SR proteins

SR proteins regulate alternative splicing in plants and are important for proteome plasticity and gene expression regulation. Moss genome annotation version 1.6 (http://www.cosmoss.org/) revealed 54 gene candidates that are potentially associated with AS processes, 18 of which were SR proteins (Table 1). Changes were analyzed in the transcription and AS of five SR proteins, related to DAS and DE genes in different life forms and treated with plant stress hormones.

**Table 1:** List of the identified serine/arginine (SR) genes.

Gene ID	Accession	Protopasts_fpkm	Gametophores_fpkm	Protonemata_fpkm	DAS/DEG	cosmoss v1.6 annotation
Pp1s31_294V6	pp-SC39	12,3687	18,9263	12,5153		serine arginine rich splicing
**Pp1s332_29V6**	pp-RSZ23	174.547	66.4998	50.4562	DEG	splicing arginine serine-rich 7
Pp1s21_218V6		80.4487	41.8722	24.6813		arginine serine-rich-splicing
Pp1s69_23V6	pp-RS2Z37	44.4132	53.8858	27.1617		arginine serine-rich splicing
**Pp1s144_89V6**	pp-RS27	80.871	145.87	43.0218	DAS	arginine serine-rich splicing factor rsp41
Pp1s65_286V6	pp-RS2Z38	28.0225	32.2262	22.8316		arginine serine-rich splicing
Pp1s3_167V6		8,88631	27,2978	11,8353		arginine serine-rich-splicing
Pp1s234_5V6		59.6123	44.5324	20.4253		arginine serine-rich-splicing
**Pp1s9_310V6**	at-RS2Z33	112,002	11,5541	12,5369	DEG/DAS	arginine serine-rich splicing
**Pp1s9_312V6**	at-RS2Z33	9.92567	2.50804	0.43477	DEG	splicing arginine serine-rich 6
Pp1s27_120V6		30.7344	38.1959	22.6916		arginine serine-rich-splicing
Pp1s169_63V6	at-SCL28	0	0.299272	0.0635878		splicing arginine serine-rich 4
Pp1s169_60V6	at-SCL28	6.12028	15.9798	8.75574		splicing arginine serine-rich 4
Pp1s270_54V6	pp-SCL33	38.4625	80.8552	31.3248		splicing arginine serine-rich 4
Pp1s207_102V6	at-RS2Z33	33.5187	25.8747	18.3497		splicing arginine serine-rich 7
Pp1s42_165V6	at-SC35	5.10322	8.84917	4.89409		serine arginine rich splicing
Pp1s183_39V6	pp-SCL42	30.0473	32.6298	30.2658		splicing arginine serine-rich 4
**Pp1s28_193V6**	pp-SR40,at-SR34a	26.4214	61.7129	14.4503	DAS	pre-mrna-splicing factor sf2

According to our data, genes Pp1s9_312V6 and Pp1s28_193V6 (SR34A homolog) differed considerably in their transcription levels (DE genes) between gametophores and protonemata, and Pp1s332_29V6 differed between protonemata and protoplasts. Pp1s9_310V6 (RSZ33) and Pp1s28_193V6 (SR34A) were DAS genes between protonemata and protoplasts, and Pp1s144_89V6 (RS27) was a DAS gene between gametophores and protonemata. RNA-seq data were validated using the RT-PCR and qRT-PCR; the level of AS of these SR genes was estimated using isoform-specific primers in each case (Supplementary Table S5, Supplementary Figure S6).

Detailed analysis of these SR genes by RT-PCR and qRT-PCR is given in Supplementary Data S2 and Supplementary Figure S7. We identified new isoforms of these genes, and data providing evidence of their translation were obtained at the transcriptome and proteome levels.

Transcription and AS of SR genes are affected by stress conditions and hormones^16, 21^. Gametophores have increased levels of LEA proteins, which are upregulated by ABA, in turn suggesting that there are elevated levels of this hormone in this life form. Previously, we showed that genes participating in the biosynthesis of jasmonic acid are upregulated in protoplasts^51^. Since this finding suggests that changes in hormonal status are associated with developmental transition or cell reprogramming, we evaluated the influence of ABA and methyl jasmonate (MeJA) on the transcription of specific isoforms of four SR genes using isoform-specific RT-PCR and qRT-PCR (Fig. 4F).

We revealed that treating protonemata with ABA and MeJA leads to changes in the transcription of the specific isoforms of two genes: Pp1s28_193V6 (SR34A homolog) and Pp1s332_29V6 (pp-RSZ23). Pp1s28_193V6 is a DAS gene in protonemata and protoplasts, and a DE gene in protonemata and gametophores. Both isoforms reacted to the treatment of protonemata with ABA in a similar way: the transcription level of each was increased. Interestingly, on treating protonemata with MeJA, the transcription level of the isoform containing a retained intron in the 5′-UTR (TCONS_00033883) increased, while no alteration was seen in the second isoform (TCONS_00030116) with AS in the 5′-UTR (Fig. 4F). This coincides with our classification of this gene as DAS in protonemata and protoplasts, and suggests a possible increase in jasmonic acid derivatives in the latter.

We also analyzed two isoforms of Pp1s332_29V6 (pp-RSZ23), which was upregulated in protoplasts (DE) by isoform-specific primers. The transcription level of both isoforms slightly increased upon treatment with ABA, but there were no changes when treated with MeJA (Fig. 4F). In this gene, no strong influence of ABA or MeJA on AS or transcription was found.

## Discussion

Alternative splicing (AS) is a pivotal mechanism for the post-transcriptional modification of eukaryotic transcripts responsible for transcriptome plasticity and cell proteome diversity^57^. In this study, we analyzed the transcriptomes of two life forms (gametophores and protonemata) and protoplasts of moss *P. patens*. We evaluated the AS of genes and identified differentially alternatively spliced (DAS) genes for which the transcriptional level of isoforms of these genes differed considerably between the different cell types. Mass spectrometry was used to evaluate the influence of AS on the cell proteome. We identified 1458 isoform-specific peptides representing 616 alternatively spliced genes. Apparently, our results indicate little correspondence between the predicted proteome plasticity shaped by AS, and the observed translation of particular isoforms at the proteomic level.

Comparing protonemata with gametophores, and protonemata with protoplasts, we found that the splicing of several hundred genes was significantly altered. Our results suggest that AS is regulated at different levels. These findings thus point to the importance of AS in responses to stress conditions and in the development of bryophytes, and seem counter to our findings that AS has no strong influence on the proteome.

### The role of alternative splicing in Reprogramming of Protoplast Cells

*P. patens* protoplasts are a promising model in which to study plant cell reprogramming since they develop into protonemal apical stem cells without needing exogenous hormones^47, 58^. In our study of protoplasts, the overrepresented GO terms associated with AS genes were those involved in transcription regulation, development, cell communication, and response to stresses. Thus, AS can play important roles in protoplasts reprogramming in moss.

In the transition from the gametophytic to the protoplast stage, expression levels of genes involved in auxin signaling are considerably elevated. For example, the genes involved in auxin polar transport – *ABCB4* (Pp1s391_45V6) and *ABCB19* (Pp1s252_67V6) – exhibited the highest transcription level on day three of gametophyte development^59^. According to our data, Pp1s252_67V6 (ppabcb16) is DAS gene; we identified several isoforms with dIF >20. Another DAS gene involved in hormone metabolism is Pp1s277_78V6 (gibberellin 3-beta-dioxygenase), the splicing of which also differed between gametophores and protonemata. In protoplasts, considerable changes were found in the transcription of two isoforms of this gene, which does not seem to correlate with proteome data. It is known that auxins and cytokinins, stimulate protoplasts from different species to reenter the cell cycle, proliferate, and undergo regenerative processes^60^. We suggest that changes in AS of genes involved in hormone signaling may stimulate protoplast reprogramming and development.

### AS influence on cell proteome is overestimated

Eukaryotic genes produce a number of alternative isoforms that are commonly believed to be a major source of cellular protein diversity^61–63^. Although many thousands of alternatively spliced transcripts were detected at the mRNA transcript level, the evidences of AS at the protein level are controversial^34, 35, 64, 65^. For instance, many of alternative isoforms are subject to cytoplasmic NMD^41, 66, 67^. Ribosome profiling and mass spectrometry analyses are two main high-throughput instruments to elucidate mRNAs translation into proteins^32, 68, 69^. Analysis of ribosome profiling data showed that at least 75% of human exon-skipping events were engaged by ribosomes^70^. However, the recent studies showed that analysis of the same ribosome profiling data might lead to controversial conclusions^71, 72^. Thus, in spite of the fact that standard mass spectrometry approaches generate relatively low proteome coverage and cannot detect peptides presented at very low levels, large-scale MS experiments are now the main source of evidence of alternative splicing at the protein level. Some of the recent proteomics studies confirmed the considerable amount of AS events at the protein level^73, 74^. However, it has been shown that these studies dramatically overestimate the number of reliable peptide identifications^75^. The other proteomic studies in species ranging from human^34, 76^ to mouse^77^, rat^78^, Drosophila^79^ and Arabidopsis^35, 36^ confirmed surprisingly small numbers of splice variants. In our study, we performed mass-spectrometry analysis to confirm splicing events in moss cells and could identify only 85 isoform-specific peptides from 25 DAS genes in our dataset. Herewith, we could only distinguish five genes that unambiguously indicated the presence of two or more splice isoforms from the same locus. The expected number of AS genes identified by *in silico* analysis was substantially larger (66 times) than those in our proteomic experiments. The reasons for low numbers of AS events, detected at the proteome level may be followed: 1) high level of false positives introduced by limitation of accuracy of instruments^80, 81^; 2) problems with reproducibility of proteomics experiments^82^; 3) challenges in correct matching of obtained spectra to peptide sequences by search engines^83^. Post-translational modifications, low-quality spectra or peptide modifications during sample preparation dramatically complicate the peptide identifications^84, 85^. Taken into account previous studies and our results, we suggest the gap between the number of isoforms detected at mRNA level and the number of proteins confirmed at proteome level is difficult to explain away as purely limitations in proteomics techniques.

On the one hand, AS can lead to the tissue-specific rewiring of protein–protein interaction networks^33^, on the other hand, stochastic processes may be responsible for creation of alternative isoforms^86^. We suggest that gene expression levels, not alternative splicing, seem to be the key to proteome diversity in moss and most alternative isoforms have little functional relevance as proteins^32^. However, there are experimental evidences supporting the tissue-specific rewiring theory and the functional role of AS^33, 87, 88^. Moreover, it has been shown that N- and C-terminal sequences of protein isoforms are enriched for intrinsically disordered regions (IDRs) and provide regulatory options for tissue-specific and conditional expression^89^. Apparently, further studies are needed to clarify a functional cellular role of alternative variants.

### Regulation of Alternative Splicing by SR Proteins

SR proteins are a family of highly conserved phosphoproteins that play a key role in regulating pre-mRNAs splicing^20^. In *A.thaliana* the splicing of SR genes is tissue-specific and is altered in different developmental stages, and by the presence of stress factors and hormones^15, 21, 90^. However, the effects of various factors on the AS of SR genes in non-vascular plants are unclear. We identified new isoforms for some SR genes, such as Pp1s144_89V6 (RS27), Pp1s9_310V6 (RS2Z33), and Pp1s332_29V6 (pp-RSZ23) (Supplementary Text S2). However, taking into account our proteomic data and known events of unproductive splicing in SR genes, we assume that the majority of these isoforms do not encode intact proteins.

When we compared gametophores and protonemata, our results suggested changes in the AS of genes involved in stress response. It may be assumed that there are specific AS regulators of these genes and, according to our results, one of these factors is the SR gene Pp1s144_89V6.2 (RS27) detected as DAS only when protonemata and gametophores are compared. A homolog of this gene regulates splicing of pre-mRNA and miRNA biogenesis in *A. thaliana* ^91^; it is also important in providing a salt-protective response, and its overexpression leads to salt-tolerance^13^. We suggest that some SR genes regulating stress-response in vascular plants play important roles in moss development.

In protoplasts and protonemata, we identified changes in AS and the transcription levels of Pp1s9_310V6 and Pp1s9_312V6, which are homologous to the SR-protein RSZ33 in Arabidopsis. Overexpression of the gene encoding the RSZ33 splicing factor in *A. thaliana* leads to phenotypic changes and auxin signaling impairment^92^. Expression of RSZ33 increases during embryogenesis, and in early stages of sporophyte development^92^. Predicted by RNA-seq, we identified two new Pp1s9_310V6 isoforms, which differed by an exon at the 5′-UTR, and a new isoform of Pp1s9_312V6. This likely indicates that this protein is important in the regulation of AS during protoplast cell reprogramming.

Upon of the cell with ABA and MeJA, we also found AS changes in Pp1s28_193V6, a homolog of the SR34A protein in *A. thaliana*. We demonstrated that, in *P. patens*, isoforms of this gene differ at the 5′-UTR. In *A. thaliana*, tissue-specific expression of At-SR34a gene isoforms and the repression by exogenous ABA were observed^21, 93^. Moreover, the At-SR34a gene is regulated by nonsense-mediated decay (NMD), which is in turn linked to splicing events at the 5′-UTR^20^. These results confirm our RNA-seq data and suggest that ABA in gametophores and MeJA in protoplasts regulate AS and the transcription of SR genes.

### AS changes lncRNA-(pre-)mRNAs interaction network

Long non-coding RNAs make up a considerable proportion of the transcriptome of eukaryotic genomes^56^. It is well known that lncRNAs are enabled of complementary binding to DNA or RNA molecules and direct transcription, post-transcriptional modifications, translation, transport and RNA degradation^94^. Therefore, the identification of lncRNA binding sites is important for understanding lncRNA based gene regulatory network.

Here we showed that the pre-mRNAs and mRNAs have thousands of potential complementary binding sites for lncRNAs. As the only small fraction of the interaction sites maintains between different isoforms, AS can be considered as one of the major regulator of lncRNA-(pre-)mRNA interaction network. In our study, 14% of the interactions are located in the alternative splicing sites. It was previously shown that antisense intronic lncRNAs may prevent splicing in the corresponding sites^95^ or change splicing of nearby exons^96^. Thus, interactions between lncRNAs and pre-mRNA splicing sites may lead to changes in AS. However, the mechanisms regulating AS in *trans* via lncRNA-mRNA interactions are poorly understood. Interaction sites identified in this study are required for further verification by downstream approaches aimed to silence lncRNAs or change the sequence of interaction sites^96^. As protocols for genome editing are well optimized for *P. patens* it can be a very suitable object for further study of functions of lncRNAs in plants.

## Material and Methods

### Protonema and Gametophore Growth and Protoplast Isolation

Protonemata and gametophores were grown as previously described^51^. To isolate RNA and protein, 5-d-old protonemata and 8-week-old gametophores were used. Protoplasts were obtained using a modified version of the previously described method^51, 97^. Protoplast sediment was frozen and used to isolate proteins and RNA.

### Sample Treatment with Hormones

Five-d-old protonemata grown on agarized Knop medium were treated with 50 mM ABA or 400 µM MeJA and incubated for 24 h under white light illuminated in a Sanyo Plant Growth Incubator MLR-352H (Panasonic, Osaka, Japan); photon flow was 61 µmol m^−2^ under a 16-h photoperiod at 24 °C. Protonemata incubated with ultrapure MilliQ water (Merck Millipore, USA) was used as the control. After incubating for 24 h, protonemata were frozen in liquid nitrogen, ground, and used for RNA isolation.

### Total RNA Isolation

Total RNA from gametophores, protonemata, and protoplasts was isolated as previously described^46^. Quality and quantity were evaluated using electrophoresis on agarose gel with ethidium bromide staining. Total RNA concentration of samples was precisely measured using the Quant-iT™ RNA Assay Kit, 5–100 ng on a Qubit 3.0 (Invitrogen, US) fluorometer.

### Reverse Transcription and Quantitative Reverse Transcription PCR

cDNA was synthesized using the MMLV RT kit (Evrogen, Russia) according to the manufacturer’s recommendations. Random hexamer primers were used to prepare cDNA from 2 µg total RNA after DNase treatment. Primers were designed using the PrimerQuest Tool (http://eu.idtdna.com/Primerquest/Home/Index) (Supplementary Table S5). For each primer pair, melting curves were analyzed to validate specificity of binding. Real-time PCR was performed using the qPCRmix-HS SYBR system and SYBR Green I (Evrogen) dye on a LightCycler® 96 (Roche, Mannheim, Germany). qPCR was carried out in three biological and three technical replicates. A minus reverse transcriptase (-RT) control contained RNA that had not been treated with reverse transcriptase to confirm the absence of DNA in samples. cDNA representation was normalized using stably transcribed AdePRT (Pp1s212_43V6.1)^98, 99^.

### Transcript Catalogue Assembly

Solid sequencing data was mapped as previously described (Fesenko *et al*.^51^). The number of uniquely mapped filtered reads was 31, 36, and 38 million for gametophore, protonema, and protoplast samples, respectively. Mapped RNA-seq data were assembled with Cufflinks v2.2.0^100^ using *Physcomitrella patens* genome version 1.6. Cuffmerge was then used to merge assemblies into a master transcriptome, using the reference sequence and reference genome specified above.

Assembly was performed using two versions of Cufflinks, v.2.2.0 and v.2.2.1. Default settings were used, plus –frag-bias-correct, and –multi-read-correct. Seven assemblies were also produced with varying –overhand-toleranсe parameters. Thus, two catalogs were obtained: set1, the Cufflinks v.2.2.0 assembly; and set 2, all transcripts occurring in more than four assemblies, generated using Cufflinks v.2.2.1 with varying –overhand-toleranсe values. Transcription levels were estimated in fragments per kilobase of exon per million fragments mapped (FPKM); only genes with FPKM ≥ 0.2 were used for further analysis. To define the functions of the identified genes, we used moss genome annotation v1.6 (http://www.cosmoss.org) and BLAST search against annotated *A. thaliana* sequences.

To define the ratio of transcripts spliced by U2 and U12 spliceosomes, we evaluated conserved splice signals in the exon–intron junctions. To do this, we considered the distribution of exons over various splice types in AS genes. Exons flanking the retained introns in the transcripts, and the first exons, were excluded from analysis. This approach identified 121 394 exons, which were further divided into groups. The Biostrings R package was used to evaluate various exon types containing conserved sequences for U2 and U12 spliceosomes. A position-probability matrix (PPM) was used to search for conserved sequences recognized by U2 and U12 spliceosome subunits.

### Differential Expression

Differential expression was calculated using Cuffdiff software included in the Cufflinks package. Subsequently, differential expression was considered significant if the *P*-value was greater than the false discovery rate (FDR) after applying the Benjamini–Hochberg correction for multiple-testing (Cuffdiff significance parameter, ‘yes’), and FDR < 0.05. The more stringent rule |log2(FPKMy/FPKMx)| > 2 was used to differentiate genes with high differential expression. To define the functions of the identified genes, we used moss genome annotation 1.6 (http://www.cosmoss.org) and BLAST search against annotated *A. thaliana* sequences.

### Differential Splicing

spliceR^101^ was used with default settings to annotate transcripts with classes of AS event. Briefly, based on the information from all transcripts originating from a single gene, spliceR constructs hypothetical pre-RNA and compares it with observed transcripts, thus classifying AS events. For each transcript in each pair (of different life forms or protoplasts), spliceR defined a delta isoform fraction (dIF) value as the difference of the contribution of a transcript to the total gene transcription level.

For all transcription start sites (TSS), Cuffdiff was used to estimate the extent of isoform switching between the isoforms originating from this TSS, as measured by the square root of the Jensen–Shannon divergence computed on the relative abundances of splice variants. Differential splicing was considered significant if *P*-value and FDR were <0.05 after applying the Benjamini–Hochberg correction for multiple testing.

For all isoforms from significantly differentially spliced TSS, the more stringent rule of absolute value of dIF above 20 was introduced in order to assess the isoforms with the highest levels of differential splicing.

### GO Term Analysis

GO groups were adopted from the *Physcomitrella patens* genome annotation version 1.6. The topGO package in the R environment was used to analyze the enrichment of GO terms in DAS, DEGs, and lncRNA–mRNA groups. Significant GO terms (*P*-value < 0.05) were selected and results visualized with REVIGO software^102^.

### Identification of Long Non-Coding RNAs

To identify lncRNA candidates, a modified CANTATAdb pipeline was used^50^. All supposed transcripts from the Cuffmerge assembly with class_code=‘u’, ‘x’, or ‘s’ were selected. Sequences of putative lncRNAs and mRNAs were excised from the genome with samtools, and reverse sequences were reversed and complemented with the revseq –reverse –complement command. The coding potential of the transcripts was evaluated as follows: transcripts over 200 nucleotide long that had not been previously annotated in genome version 1.6 were selected and analyzed for their coding potential using the CNCI program (https://github.com/www-bioinfo-org/CNCI) and Rfam database. Using this approach, transcripts with coding potential (CNCI <0), those with plastid homology (nine transcripts), and mitochondrial genomes (not found) were excluded. To improve the reliability of the data, lncRNAs with FPKM less than 0.5 were discarded in all three cell types. To avoid known RNAs, BLASTN was used on the Rfam plant base; at this stage, the selection criteria were: e-value < 0.00001 and p.ident >97%. Sequences remaining after the filtration stages were used for further analysis as predicted lncRNAs.

Antisense lncRNA may play an important role in the regulation of gene expression. Therefore, we identified cis-natural antisense transcripts (NATs) using the information on overlapping between protein-coding and lncRNAs genes. Eighty-eight lncRNAs were transcribed as cis-NAT, and overlapped with 86 loci of genome version 1.6 (Supplemental Table S4, List 1).

### Identification of lncRNA–RNA Interactions

The LAST alignment tool was used since it allows a custom score matrix to be utilized^103^; this is important because of the presence of so-called ‘wobble pairs’ in addition to canonical compounds. The following values were used in our score matrix: G:C = 4, A:T = 2, G:T = 1; all other cases were termed –6. lncRNAs (query) were aligned on mRNA (database) with the parameters –Q0 –a20 –m100 –s0 –b8, and the –e parameter was evaluated using the lastex tool for each alignment^103^. For further analysis, lncRNA transcripts with an expression level >0.5 FPKM were used, and mRNAs with an expression level >1 FPKM, in at least one sample.

### *In silico* AS detection experiment

We performed an *in silico* simulation to estimate the number of AS genes we would expect to detect in the experiments as described previously^34^. For the simulation, we took genes that were identified in our MS data and assumed that all protein isoforms were expressed equally. We generated tryptic peptides from custom database of predicted ORFs by *in silico* trypsinolysis. Then we selected at random the same number of peptides for each gene as we identified in our MS data. We calculated the number of genes for which two or more protein isoforms can be unambiguously distinguished by found isoform specific peptides. This procedure was repeated 100 times.

### Protein Extraction and Trypsinolysis

Proteins were isolated using the phenol extraction method as previously described^104^. Four biological repeats for gametophores, four for protonemal and two for protoplast samples were used.

### LC-MS/MS Analysis

Tryptic peptides were analyzed on a TripleTOF 5600+ (ABSciex, Canada) quadrupole time-of-flight mass spectrometer equipped with a NanoSpray III ion source combined with a NanoLC Ultra 2D+ nano-HPLC (Eksigent) system as previously described^51^.

Protein database was generated by TransDecoder (http://transdecoder.sourceforge.net/) from the assembled transcripts. TransDecoder was also used for a homology search with the available protein sequences and presence of the known domains using Uniref90 and pfam databases respectively. Raw LC-MS/MS data were converted to peaklists with ProteinPilot 4.5 revision 1656 (Sciex, Canada) using a standard set of identification settings over the protein database. To identify proteins, peaklists were analyzed with MASCOT (version 2.2.07) and X! Tandem (CYCLONE, 2013.2.01) search engines against the protein database with the concatenated reverse decoy dataset. The precursor and fragment mass tolerance were set at 20 ppm and 0.04 Da, respectively. Database-searching parameters included the following: tryptic digestion with one possible missed cleavage, static modifications for carbamidomethyl (C). For X! Tandem we also selected parameters that allowed a quick check for protein N-terminal residue acetylation, peptide N-terminal glutamine ammonia loss, or peptide N-terminal glutamic acid water loss. Result files were submitted to the Scaffold 4 software (version 4.0.7) for validation and meta-analysis. We used the local false discovery rate scoring algorithm with standard experiment-wide protein grouping. For the evaluation of peptide and protein hits, a false discovery rate of 5% was selected for both. False positive identifications were based on reverse database analysis.

### Availability of Supporting Data

The mass spectrometry proteomics data have been deposited to the ProteomeXchange Consortium via the PRIDE^105^ partner repository with the dataset identifier PXD005223 and 10.6019/PXD005223.

## Electronic supplementary material

Supplementary Information

Supplementary Table S1

Supplementary Table S2

Supplementary Table S3

Supplementary Table S4

Supplementary Table S5

## References

[1] Mastrangelo AM, Marone D, Laido G, De Leonardis AM, De Vita P (2012). Alternative splicing: enhancing ability to cope with stress via transcriptome plasticity. Plant science: an international journal of experimental plant biology.

[2] Staiger D, Brown JW (2013). Alternative splicing at the intersection of biological timing, development, and stress responses. The Plant cell.

[3] Filichkin S, Priest HD, Megraw M, Mockler TC (2015). Alternative splicing in plants: directing traffic at the crossroads of adaptation and environmental stress. Current opinion in plant biology.

[4] Thatcher SR (2016). Genome-Wide Analysis of Alternative Splicing during Development and Drought Stress in Maize. Plant physiology.

[5] Filichkin SA (2010). Genome-wide mapping of alternative splicing in Arabidopsis thaliana. Genome research.

[6] Zhang G (2010). Deep RNA sequencing at single base-pair resolution reveals high complexity of the rice transcriptome. Genome research.

[7] Marquez Y, Brown JW, Simpson C, Barta A, Kalyna M (2012). Transcriptome survey reveals increased complexity of the alternative splicing landscape in Arabidopsis. Genome research.

[8] Li Q, Xiao G, Zhu YX (2014). Single-nucleotide resolution mapping of the Gossypium raimondii transcriptome reveals a new mechanism for alternative splicing of introns. Molecular plant.

[9] Shen Y (2014). Global dissection of alternative splicing in paleopolyploid soybean. The Plant cell.

[10] Egawa C (2006). Differential regulation of transcript accumulation and alternative splicing of a DREB2 homolog under abiotic stress conditions in common wheat. Genes & genetic systems.

[11] Tanabe N, Yoshimura K, Kimura A, Yabuta Y, Shigeoka S (2007). Differential expression of alternatively spliced mRNAs of Arabidopsis SR protein homologs, atSR30 and atSR45a, in response to environmental stress. Plant & cell physiology.

[12] Gassmann W (2008). Alternative splicing in plant defense. Current topics in microbiology and immunology.

[13] Ding F (2014). Genome-wide analysis of alternative splicing of pre-mRNA under salt stress in Arabidopsis. BMC genomics.

[14] Li W, Lin WD, Ray P, Lan P, Schmidt W (2013). Genome-wide detection of condition-sensitive alternative splicing in Arabidopsis roots. Plant physiology.

[15] Duque P (2011). A role for SR proteins in plant stress responses. Plant signaling & behavior.

[16] Reddy AS, Marquez Y, Kalyna M, Barta A (2013). Complexity of the alternative splicing landscape in plants. The Plant cell.

[17] Vitulo N (2014). A deep survey of alternative splicing in grape reveals changes in the splicing machinery related to tissue, stress condition and genotype. BMC plant biology.

[18] Day IS (2012). Interactions of SR45, an SR-like protein, with spliceosomal proteins and an intronic sequence: insights into regulated splicing. The Plant journal: for cell and molecular biology.

[19] Thomas J (2012). Identification of an intronic splicing regulatory element involved in auto-regulation of alternative splicing of SCL33 pre-mRNA. The Plant journal: for cell and molecular biology.

[20] Rauch HB (2014). Discovery and expression analysis of alternative splicing events conserved among plant SR proteins. Molecular biology and evolution.

[21] Palusa SG, Ali GS, Reddy AS (2007). Alternative splicing of pre-mRNAs of Arabidopsis serine/arginine-rich proteins: regulation by hormones and stresses. The Plant journal: for cell and molecular biology.

[22] Kornienko AE, Guenzl PM, Barlow DP, Pauler FM (2013). Gene regulation by the act of long non-coding RNA transcription. BMC biology.

[23] Bardou F (2014). Long noncoding RNA modulates alternative splicing regulators in Arabidopsis. Developmental cell.

[24] Geisler S, Coller J (2013). RNA in unexpected places: long non-coding RNA functions in diverse cellular contexts. Nature reviews. Molecular cell biology.

[25] Szczesniak MW, Makalowska I (2016). lncRNA-RNA Interactions across the Human Transcriptome. PloS one.

[26] Terai G, Iwakiri J, Kameda T, Hamada M, Asai K (2016). Comprehensive prediction of lncRNA-RNA interactions in human transcriptome. BMC genomics.

[27] Katayama S (2005). Antisense transcription in the mammalian transcriptome. Science.

[28] Morrissy AS, Griffith M, Marra MA (2011). Extensive relationship between antisense transcription and alternative splicing in the human genome. Genome research.

[29] Wang H (2014). Genome-wide identification of long noncoding natural antisense transcripts and their responses to light in Arabidopsis. Genome research.

[30] Mollet IG (2010). Unconstrained mining of transcript data reveals increased alternative splicing complexity in the human transcriptome. Nucleic acids research.

[31] Uhlen M (2015). Proteomics. Tissue-based map of the human proteome. Science.

[32] Tress ML, Abascal F, Valencia A (2016). Alternative Splicing May Not Be the Key to Proteome Complexity. Trends in biochemical sciences.

[33] Yang X (2016). Widespread Expansion of Protein Interaction Capabilities by Alternative Splicing. Cell.

[34] Abascal F (2015). Alternatively Spliced Homologous Exons Have Ancient Origins and Are Highly Expressed at the Protein Level. PLoS computational biology.

[35] Severing EI, van Dijk AD, van Ham RC (2011). Assessing the contribution of alternative splicing to proteome diversity in Arabidopsis thaliana using proteomics data. BMC plant biology.

[36] Castellana NE (2008). Discovery and revision of Arabidopsis genes by proteogenomics. Proceedings of the National Academy of Sciences of the United States of America.

[37] Severing EI, van Dijk AD, Stiekema WJ, van Ham RC (2009). Comparative analysis indicates that alternative splicing in plants has a limited role in functional expansion of the proteome. BMC genomics.

[38] Zhu Y (2014). SpliceVista, a tool for splice variant identification and visualization in shotgun proteomics data. Molecular & cellular proteomics: MCP.

[39] Stastna M, Van Eyk JE (2012). Analysis of protein isoforms: can we do it better?. Proteomics.

[40] Brown JW (2015). Lost in Translation: Pitfalls in Deciphering Plant Alternative Splicing Transcripts. The Plant cell.

[41] Kalyna M (2012). Alternative splicing and nonsense-mediated decay modulate expression of important regulatory genes in Arabidopsis. Nucleic acids research.

[42] Schweingruber C, Rufener SC, Zund D, Yamashita A, Muhlemann O (2013). Nonsense-mediated mRNA decay - mechanisms of substrate mRNA recognition and degradation in mammalian cells. Biochimica et biophysica acta.

[43] Gonzalez-Porta M, Frankish A, Rung J, Harrow J, Brazma A (2013). Transcriptome analysis of human tissues and cell lines reveals one dominant transcript per gene. Genome biology.

[44] Rensing SA (2008). The Physcomitrella genome reveals evolutionary insights into the conquest of land by plants. Science.

[45] Prigge MJ, Bezanilla M (2010). Evolutionary crossroads in developmental biology: Physcomitrella patens. Development.

[46] Cove, D. J. *et al*. The moss Physcomitrella patens: a novel model system for plant development and genomic studies. *Cold Spring Harbor protocols* **2009**, pdb emo115, doi:10.1101/pdb.emo115 (2009). 10.1101/pdb.emo115 20147063

[47] Cove, D. J. *et al*. Isolation and regeneration of protoplasts of the moss Physcomitrella patens. *Cold Spring Harbor protocols* **2009**, pdb prot5140, doi:10.1101/pdb.prot5140 (2009). 10.1101/pdb.prot5140 20147070

[48] Wu HP (2014). Genome-wide analysis of light-regulated alternative splicing mediated by photoreceptors in Physcomitrella patens. Genome biology.

[49] Chang CY, Lin WD, Tu SL (2014). Genome-Wide Analysis of Heat-Sensitive Alternative Splicing in Physcomitrella patens. Plant physiology.

[50] Szczesniak MW, Rosikiewicz W, Makalowska I (2016). CANTATAdb: A Collection of Plant Long Non-Coding RNAs. Plant & cell physiology.

[51] Fesenko IA (2015). Specific pools of endogenous peptides are present in gametophore, protonema, and protoplast cells of the moss Physcomitrella patens. BMC plant biology.

[52] Simpson CG, Brown JW (2008). U12-dependent intron splicing in plants. Current topics in microbiology and immunology.

[53] Sharp PA, Burge CB (1997). Classification of introns: U2-type or U12-type. Cell.

[54] Kramer M (2013). Alternative 5′ untranslated regions are involved in expression regulation of human heme oxygenase-1. PloS one.

[55] Hughes TA (2006). Regulation of gene expression by alternative untranslated regions. Trends in genetics: TIG.

[56] Iyer MK (2015). The landscape of long noncoding RNAs in the human transcriptome. Nature genetics.

[57] Roy B, Haupt LM, Griffiths LR (2013). Review: Alternative Splicing (AS) of Genes As An Approach for Generating Protein Complexity. Current genomics.

[58] Wang X (2014). The phosphoproteome in regenerating protoplasts from Physcomitrella patens protonemata shows changes paralleling postembryonic development in higher plants. Journal of experimental botany.

[59] Xiao L, Wang H, Wan P, Kuang T, He Y (2011). Genome-wide transcriptome analysis of gametophyte development in Physcomitrella patens. BMC plant biology.

[60] Xiao L, Zhang L, Yang G, Zhu H, He Y (2012). Transcriptome of protoplasts reprogrammed into stem cells in Physcomitrella patens. PloS one.

[61] Djebali S (2012). Landscape of transcription in human cells. Nature.

[62] Pan Q, Shai O, Lee LJ, Frey BJ, Blencowe BJ (2008). Deep surveying of alternative splicing complexity in the human transcriptome by high-throughput sequencing. Nature genetics.

[63] Nilsen TW, Graveley BR (2010). Expansion of the eukaryotic proteome by alternative splicing. Nature.

[64] Mele M (2015). Human genomics. The human transcriptome across tissues and individuals. Science.

[65] Kelemen O (2013). Function of alternative splicing. Gene.

[66] Yap K, Lim ZQ, Khandelia P, Friedman B, Makeyev EV (2012). Coordinated regulation of neuronal mRNA steady-state levels through developmentally controlled intron retention. Genes & development.

[67] Wong JJ (2013). Orchestrated intron retention regulates normal granulocyte differentiation. Cell.

[68] Ingolia NT, Ghaemmaghami S, Newman JR, Weissman JS (2009). Genome-wide analysis *in vivo* of translation with nucleotide resolution using ribosome profiling. Science.

[69] Ingolia NT (2016). Ribosome Footprint Profiling of Translation throughout the Genome. Cell.

[70] Weatheritt RJ, Sterne-Weiler T, Blencowe BJ (2016). The ribosome-engaged landscape of alternative splicing. Nature structural & molecular biology.

[71] Ruiz-Orera J, Messeguer X, Subirana JA, Alba MM (2014). Long non-coding RNAs as a source of new peptides. eLife.

[72] Guttman M, Russell P, Ingolia NT, Weissman JS, Lander ES (2013). Ribosome profiling provides evidence that large noncoding RNAs do not encode proteins. Cell.

[73] Wilhelm M (2014). Mass-spectrometry-based draft of the human proteome. Nature.

[74] Kim MS (2014). A draft map of the human proteome. Nature.

[75] Ezkurdia I, Vazquez J, Valencia A, Tress M (2014). Analyzing the first drafts of the human proteome. Journal of proteome research.

[76] Ezkurdia I (2015). Most highly expressed protein-coding genes have a single dominant isoform. Journal of proteome research.

[77] Brosch M (2011). Shotgun proteomics aids discovery of novel protein-coding genes, alternative splicing, and “resurrected” pseudogenes in the mouse genome. Genome research.

[78] Low TY (2013). Quantitative and qualitative proteome characteristics extracted from in-depth integrated genomics and proteomics analysis. Cell reports.

[79] Tress ML, Bodenmiller B, Aebersold R, Valencia A (2008). Proteomics studies confirm the presence of alternative protein isoforms on a large scale. Genome biology.

[80] Resing KA (2004). Improving reproducibility and sensitivity in identifying human proteins by shotgun proteomics. Analytical chemistry.

[81] Dodds ED, Clowers BH, Hagerman PJ, Lebrilla CB (2008). Systematic characterization of high mass accuracy influence on false discovery and probability scoring in peptide mass fingerprinting. Analytical biochemistry.

[82] Zhang F, Chen JY (2016). A method for identifying discriminative isoform-specific peptides for clinical proteomics application. BMC genomics.

[83] Chen Y, Zhang J, Xing G, Zhao Y (2009). Mascot-derived false positive peptide identifications revealed by manual analysis of tandem mass spectra. Journal of proteome research.

[84] Chick JM (2015). A mass-tolerant database search identifies a large proportion of unassigned spectra in shotgun proteomics as modified peptides. Nature biotechnology.

[85] Bogdanow B, Zauber H, Selbach M (2016). Systematic Errors in Peptide and Protein Identification and Quantification by Modified Peptides. Molecular & cellular proteomics: MCP.

[86] Melamud E, Moult J (2009). Stochastic noise in splicing machinery. Nucleic acids research.

[87] Buljan M (2012). Tissue-specific splicing of disordered segments that embed binding motifs rewires protein interaction networks. Molecular cell.

[88] Ellis JD (2012). Tissue-specific alternative splicing remodels protein-protein interaction networks. Molecular cell.

[89] Shabalina SA, Ogurtsov AY, Spiridonov NA, Koonin EV (2014). Evolution at protein ends: major contribution of alternative transcription initiation and termination to the transcriptome and proteome diversity in mammals. Nucleic acids research.

[90] Isshiki M, Tsumoto A, Shimamoto K (2006). The serine/arginine-rich protein family in rice plays important roles in constitutive and alternative splicing of pre-mRNA. The Plant cell.

[91] Chen T, Cui P, Xiong L (2015). The RNA-binding protein HOS5 and serine/arginine-rich proteins RS40 and RS41 participate in miRNA biogenesis in Arabidopsis. Nucleic acids research.

[92] Kalyna M, Lopato S, Barta A (2003). Ectopic expression of atRSZ33 reveals its function in splicing and causes pleiotropic changes in development. Molecular biology of the cell.

[93] Cruz TM, Carvalho RF, Richardson DN, Duque P (2014). Abscisic acid (ABA) regulation of Arabidopsis SR protein gene expression. International journal of molecular sciences.

[94] Mercer TR, Dinger ME, Mattick JS (2009). Long non-coding RNAs: insights into functions. Nature reviews. Genetics.

[95] Beltran M (2008). A natural antisense transcript regulates Zeb2/Sip1 gene expression during Snail1-induced epithelial-mesenchymal transition. Genes & development.

[96] Hu S, Wang X, Shan G (2016). Insertion of an Alu element in a lncRNA leads to primate-specific modulation of alternative splicing. Nature structural & molecular biology.

[97] Liu, Y. C. & Vidali, L. Efficient polyethylene glycol (PEG) mediated transformation of the moss Physcomitrella patens. *Journal of visualized experiments*: *JoVE*, doi:10.3791/2560 (2011). 10.3791/2560 PMC3169274 21540817

[98] Le Bail A, Scholz S, Kost B (2013). Evaluation of reference genes for RT qPCR analyses of structure-specific and hormone regulated gene expression in Physcomitrella patens gametophytes. PloS one.

[99] Pfaffl MW (2001). A new mathematical model for relative quantification in real-time RT-PCR. Nucleic acids research.

[100] Trapnell C (2012). Differential gene and transcript expression analysis of RNA-seq experiments with TopHat and Cufflinks. Nature protocols.

[101] Vitting-Seerup K, Porse BT, Sandelin A, Waage J (2014). spliceR: an R package for classification of alternative splicing and prediction of coding potential from RNA-seq data. BMC bioinformatics.

[102] Supek F, Bosnjak M, Skunca N, Smuc T (2011). REVIGO summarizes and visualizes long lists of gene ontology terms. PloS one.

[103] Kielbasa SM, Wan R, Sato K, Horton P, Frith MC (2011). Adaptive seeds tame genomic sequence comparison. Genome research.

[104] Fesenko, I. *et al*. The Physcomitrella patens Chloroplast Proteome Changes in Response to Protoplastation. *Frontiers in plant science* **7**, doi:10.3389/fpls.2016.01661 (2016). 10.3389/fpls.2016.01661 PMC5095126 27867392

[105] Vizcaino, J. A. *et al*. update of the PRIDE database and its related tools. *Nucleic acids research*, doi:10.1093/nar/gkw880 (2016). 10.1093/nar/gkw880 PMC5159556 27683222

