# Supplementary material for: Alternative splicing shapes transcriptome but not proteome diversity in Physcomitrella patens
Source: Sci Rep. 2017 Jun 2;7:2698. doi: 10.1038/s41598-017-02970-z (PMC5457400; doi:10.1038/s41598-017-02970-z)
Supplement: Supplementary file 1 — Supplementary Information [file 41598_2017_2970_MOESM1_ESM.pdf]

Alternative splicing shapes transcriptome but not proteome diversity in *Physcomitrella patens*

Igor Fesenko[1,*], Regina Khazigaleeva[1], Ilya Kirov[1], Andrey Kniazev[1], Oksana Glushenko[2], Konstantin Babalyan[1], Georgij Arapidi[1], Tatyana Shashkova[2], Ivan Butenko[2], Victor Zgoda[3], Ksenia Anufrieva[1], Anna Seredina[1], Anna Filippova[1] and Vadim Govorun[1,2]

[1]Laboratory of Proteomics, Shemyakin and Ovchinnikov Institute of Bioorganic Chemistry, Russian Academy of Sciences, Moscow, Russia

[2]Laboratory of the Proteomic Analysis, Research Institute for Physico-Chemical Medicine, Moscow, Russia

[3]Institute of Biomedical Chemistry, Moscow, Russian Federation

* To whom correspondence should be addressed. Tel: +79163237492; Email: fesigor@gmail.com

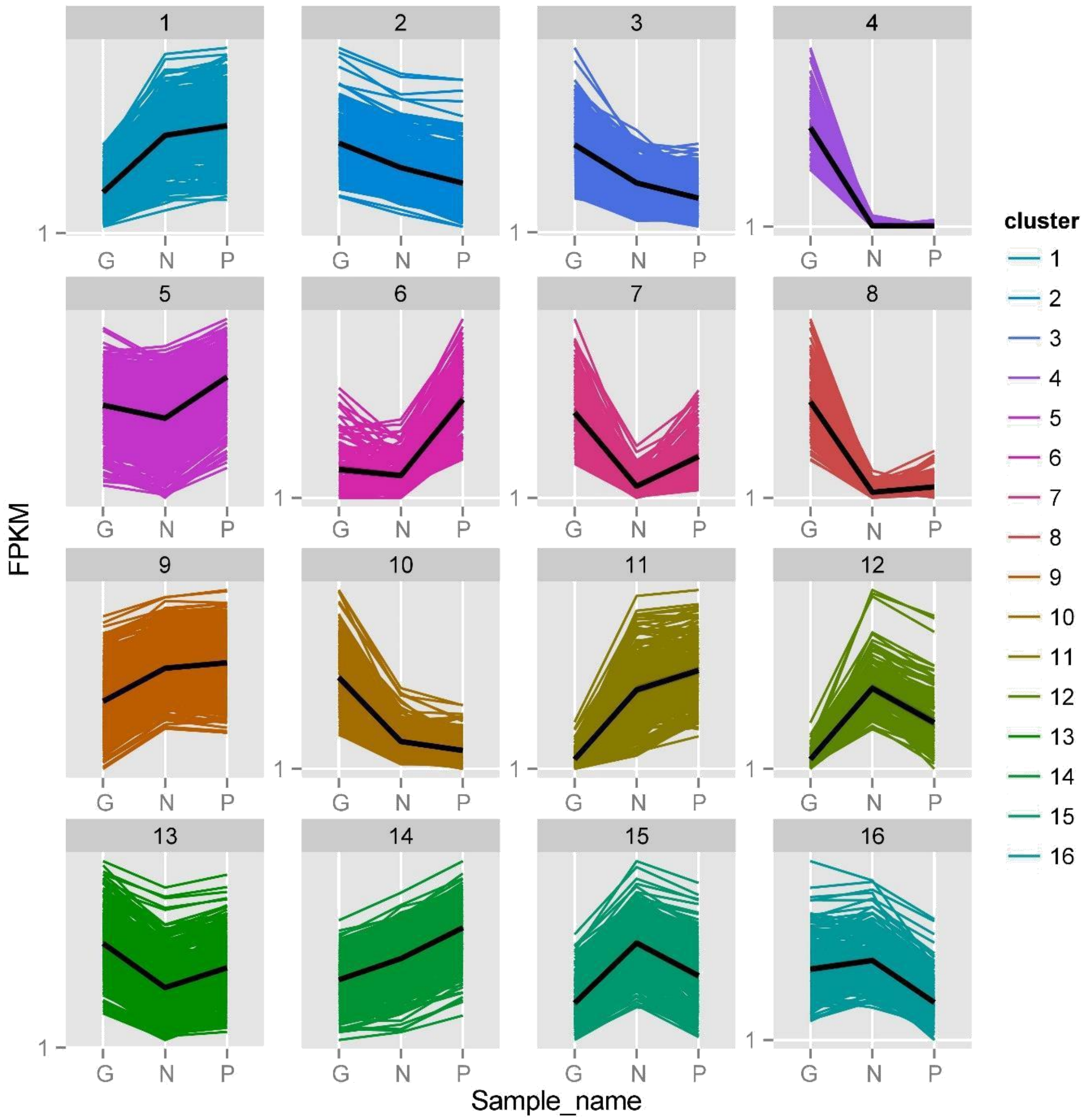

Supplementary Figure S1. Analysis of gene expression using k-means clustering in gametophores, protonema and protoplasts. Differentially expressed (DE) genes of gametophores, protonema and protoplasts were divided into 16 clusters using the k-means method. The X-axis represents life stages, divided into G: gametophores; N: protonemata; and P: protoplasts. The Y-axis represents the fragments per kilobase of transcript per million mapped reads (FPKM) value of DE genes.

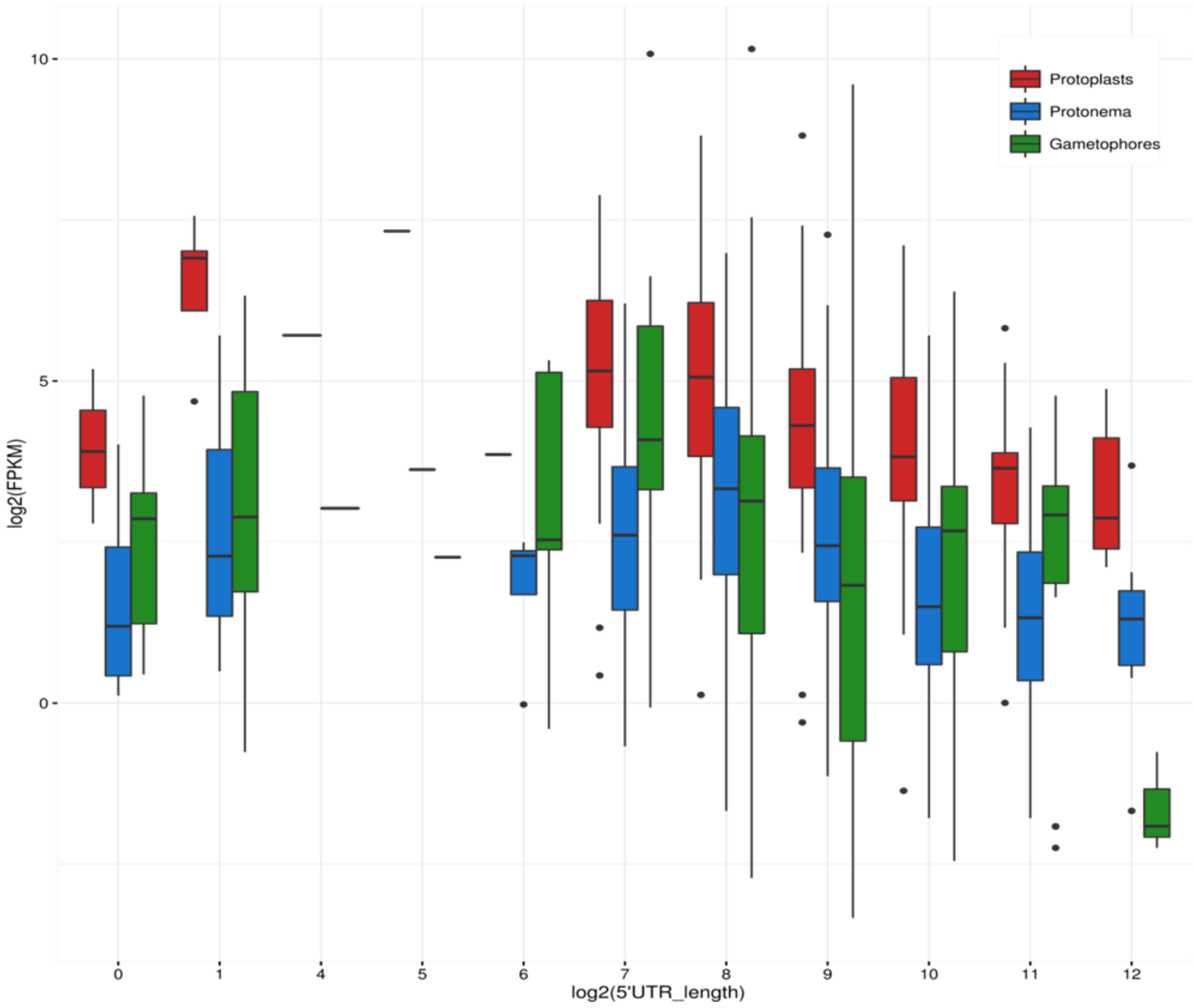

Supplemental Figure S2. Box plot showing the effect of transcripts' 5'-UTR sequence length on the level of expression of corresponding *P. patens* gene transcripts. P-protoplasts; N- protonema; G-gametophores. The Y-axis represents the FPKM value. The X-axis represents the 5'-UTR length.

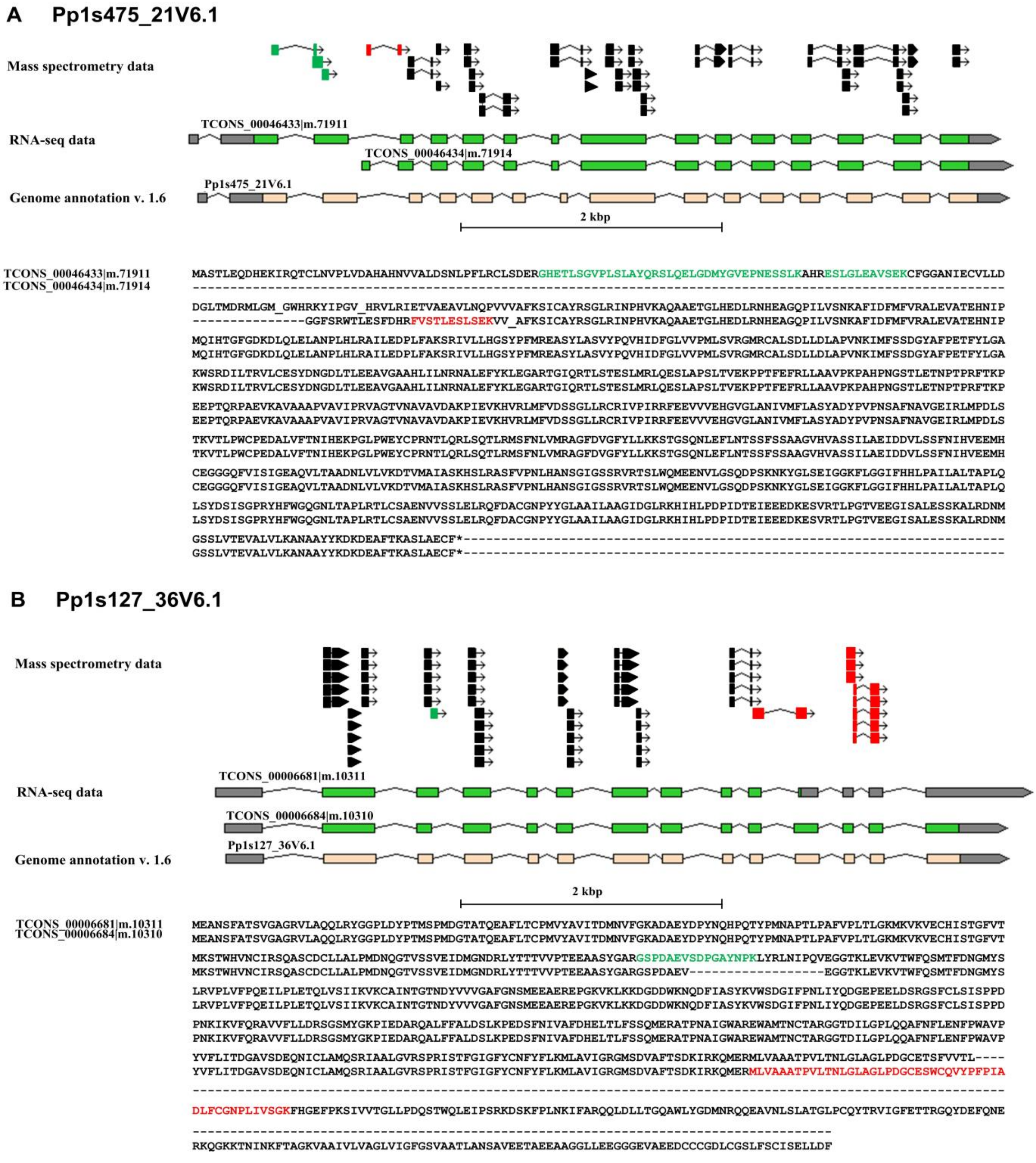

Supplemental Figure S3. Examples of alternative splicing events for two moss genes. Schemes from genome browser and pairwise alignments between two isoforms of moss genes Pp1s475_21V6 (**A**) and Pp1s127_36V6 (**B**). Predicted ORFs in RNA-seq data lines are shown in green. Identified tryptic peptides are shown in black (if they do not discriminate between the two isoforms). Isoform specific peptides are shown in red and green.

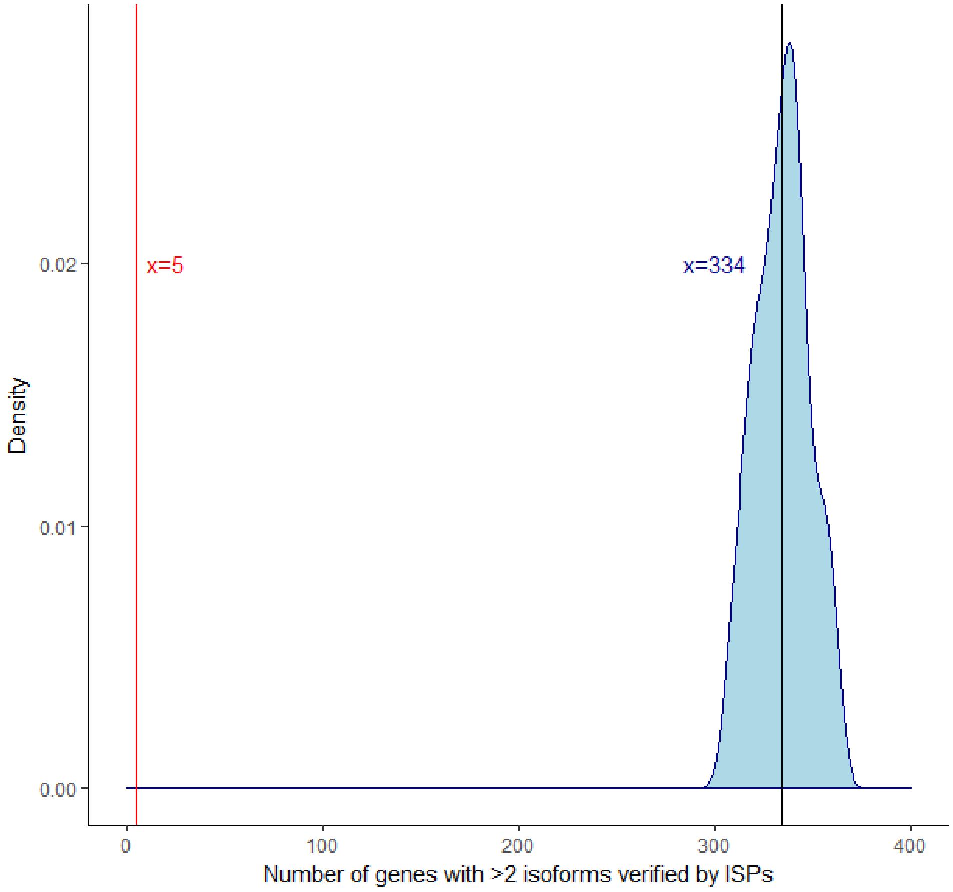

Supplemental Figure S4. The distribution of the number of AS genes with two or more protein isoforms from the *in silico* simulation after 100 iterations (density plot) and obtained from our MS data (red line). The average value is indicated for simulation experiments.

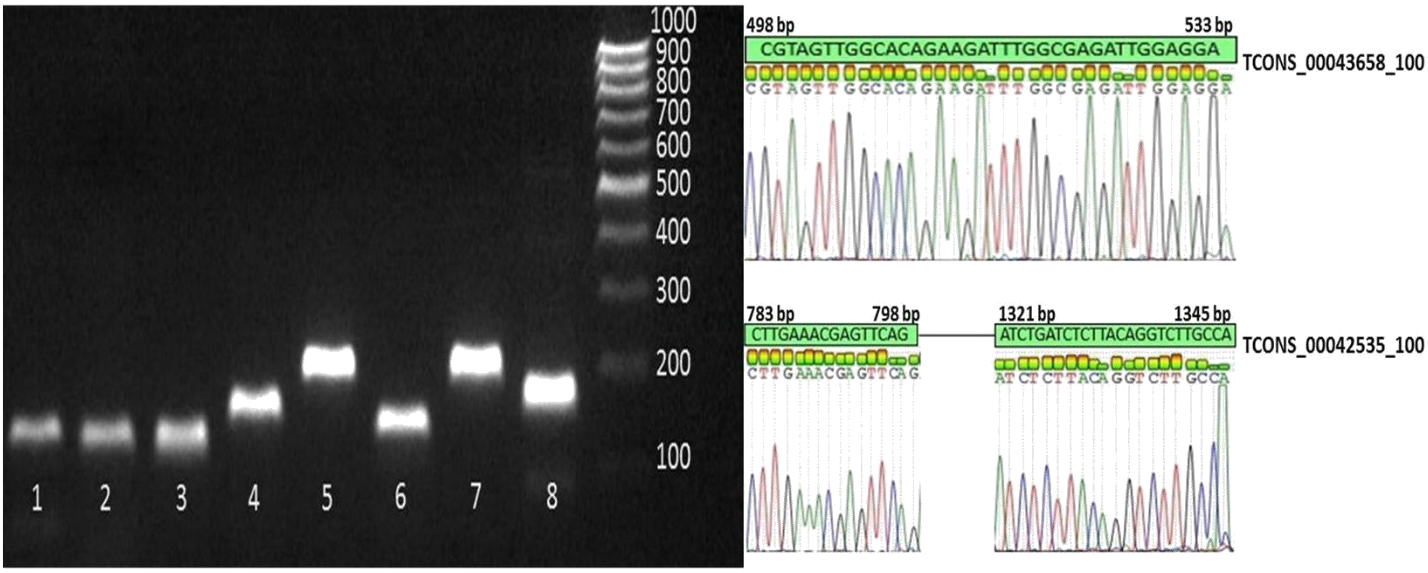

Supplemental Figure S5. Agarose gel showing RT-PCR products of the predicted lncRNAs and the sequences of two of them for an extra validation. 1. TCONS_00002301_100; 2. TCONS_00001049_100; 3. TCONS_00057154_100; 4.TCONS_00034225_100; 5. TCONS_00036127_100; 6. TCONS_00073591_25; 7. TCONS_00043658_100; 8. TCONS_00042535_100.

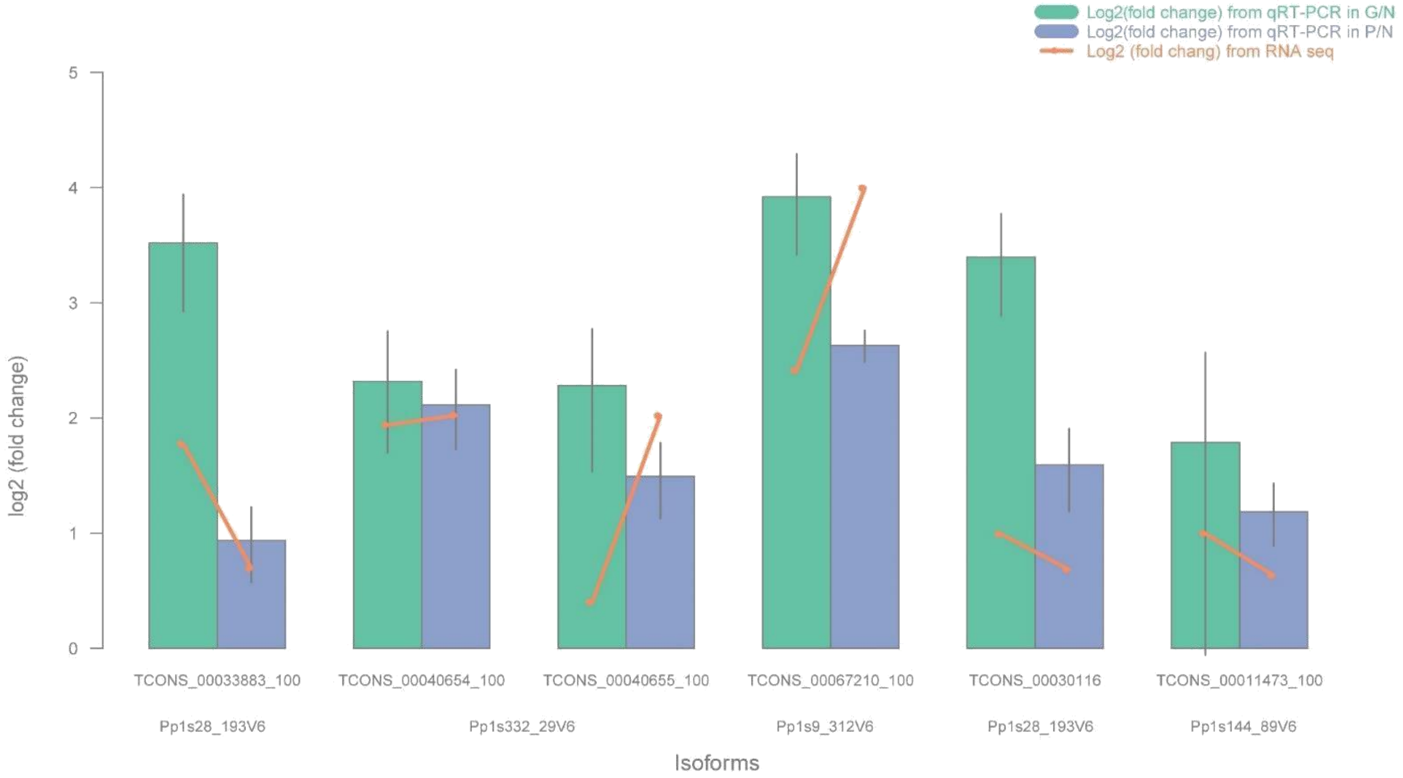

Supplemental Figure S6. Bar chart showing the results of validation of RNA-seq data by quantitative Reverse Transcription PCR (qRT-PCR) and reverse transcription PCR (RT-PCR) for the SR genes: Pp1s28_193V6, Pp1s332_29V6, Pp1s9_312V6, Pp1s144_89V6.

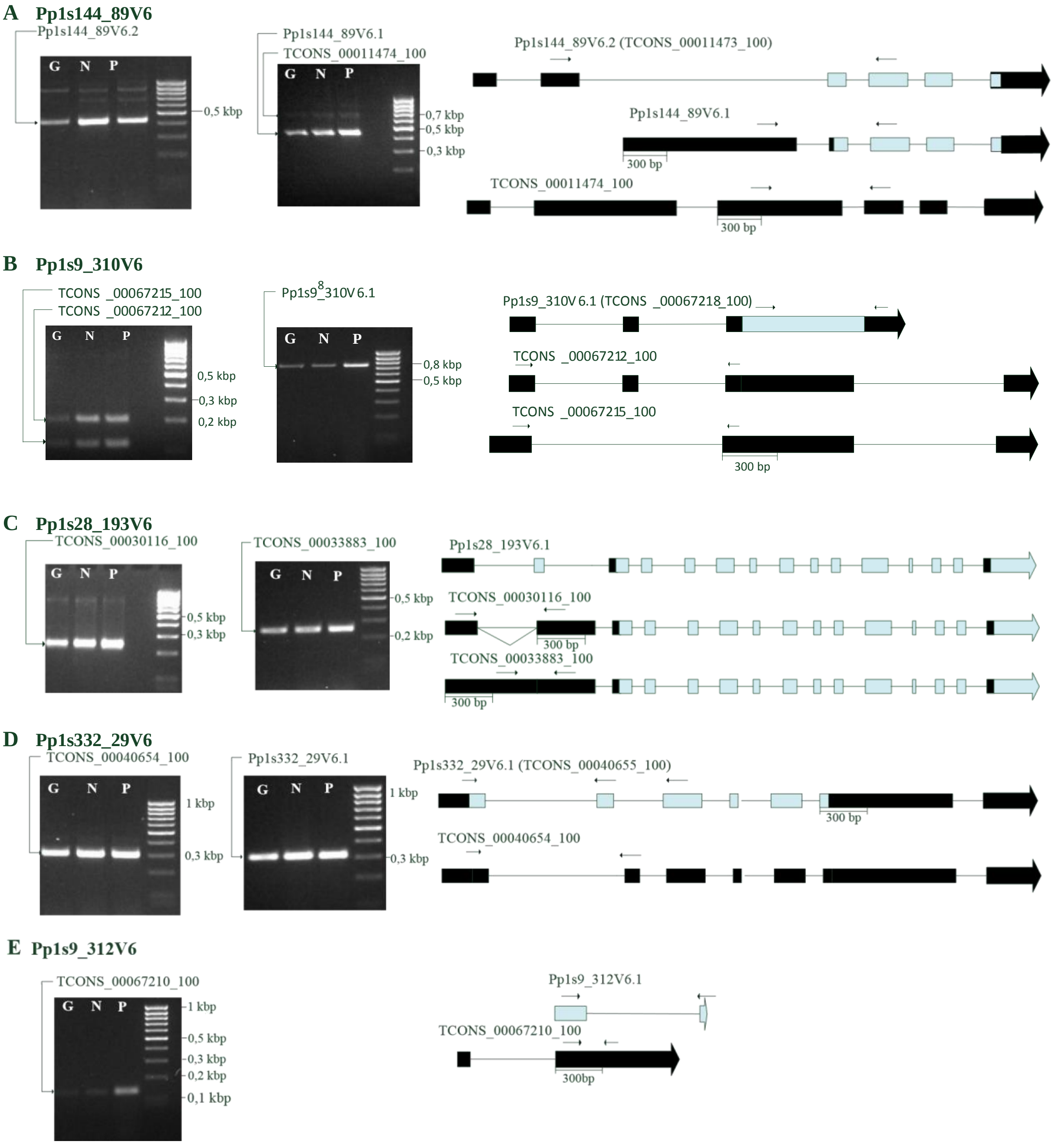

Supplemental Figure S7. The expression analysis of the SR genes: Pp1s144_89V6 (A), Pp1s9_310V6 (B), Pp1s28_193V6 (C), Pp1s332_29V6 (D), Pp1s9_312V6 (E). Panel A – E display RT-PCR products of SR genes from resolved on agarose gels on the left side and the gene structure of the mRNA isoforms on the right side. Arrows indicate the primers used for the amplification of PCR products.

Supplementary Data S1. Analysis of Differentially Expressed Genes in Protonema, Gametophore, and Protoplast Cells

We compared the transcription level of DE genes in protonemata, gametophores, and protoplasts using k-medians clustering (Supplementary Figure S1; Supplementary Table S1). Terms in the Gene Ontology (GO) annotation were used for functional analysis.

The transcript accumulation of genes in clusters 6 and 14 (Supplementary Figure S1) was increased in protoplasts. In cluster 6, transcription factors such as Pp1s259_102V6 (homeobox protein 21), Pp1s73_109V6 (myb-like HTH transcriptional regulator family protein), and Pp1s37_353V6 (MADS-box transcription factor family protein) were identified. In addition to transcription factors, we detected stress response genes including Pp1s300_55V6 (heat shock protein 101) and Pp1s322_34V6 ERD (early responsive to dehydration stress family protein). As shown in Supplementary Figure S1 in clusters 8 and 10 we identified stress response genes such as Pp1s127_22V6, (early responsive to dehydration stress protein, ERD4), Pp1s23_265V6 (SAUR-like auxin-responsive protein family), and Pp1s75_234V6 (phosphate deficiency response 2 protein). Interestingly, these clusters included regulatory genes such as Pp1s199_87V6 (cycling DOF factor 2), Pp1s882_1V6 (GRAS family transcription factor), Pp1s4_109V6 (lectin protein kinase family protein), Pp1s252_49V6 (CHASE domain-containing histidine kinase protein), and Pp1s238_70V6 (myb domain protein 33). In cluster 15, we identified transcription factors such as Pp1s7_383V6 (AP2/B3-like transcriptional factor family protein), Pp1s36_177V6 (cyclin-dependent kinase B1;2), Pp1s63_198V6 (GRAS family transcription factor), and protein kinase superfamily proteins AT1G62400, Pp1s642_3V6 (cyclin A1), Pp1s89_130V6 (CYCLIN B2;4), and Pp1s24_216V6 (CYCLIN D1;1). Increased expression of transcription factors belonging to the AP2/ERF (Pp1s6_75V6 and others), NAC (Pp1s140_93V6 and others), and WRKY (Pp1s158_166V6 and others) families was also revealed in protoplasts; these transcription factors generally regulate the expression of genes involved in growth and development processes, as well as responses to abiotic and biotic stresses. The expression of transcription factors containing the B3 domain (Pp1s167_8V6) decreased; this family of proteins participates in the regulation of auxin-dependent gene functions.

In protoplasts, as opposed to protonemata, expression levels considerably increased for Pp1s41_314V6 AOC2 (allene oxide cyclase 2), Pp1s135_3V6 AOC4 (allene oxide cyclase 4), and Pp1s97_112V6 AOS (allene oxide synthase); these genes fall in GO categories GO:0009695 and GO:0009694, indicating processes involved in the biosynthesis and metabolism, respectively, of jasmonic acid – a stress hormone activated in response to biotic stress.

Supplementary Data S2. Analysis of differentially alternatively spliced serine/arginine-rich (SR) genes.

Pp1s144_89V6 (RS27) belongs to the arginine/serine-rich (RS) subfamily of SR proteins, which are unique to plants, and encodes two isoforms (according to genome annotation 1.6). In *P. patens* the second isoform may carry a premature termination codon (PTC) or encode a protein lacking the RRM at its 5' end. The isoform lacking the RRM domain is conserved in maize, sorghum, Arabidopsis, and moss [1]. We confirmed the presence of an isoform of this protein with two RRM domains in *P. patens* gametophore, protonema, and protoplast proteomes by analysis with mass spectrometry (Supplementary Table S3). Using RT-PCR and qRT-PCR, two annotated mRNA isoforms differing at their 5'-UTRs were identified, Pp1s144_89V6.1 and Pp1s144_89V6.2; a single new isoform was also predicted by RNA-seq data, and differed from the Pp1s144_89V6.1 isoform by the incorporation of the intron into the second exon (Supplementary Figure S7A).

Pp1s9_310V6 (RS2Z33) belongs to the RSZ subfamily of SR proteins, and differs by the presence of an AS intron incorporating part of the protein's RRM1 domain [1]. With RT-PCR, the transcription of two new isoforms (TCONS_00067212_100 and TCONS_00067215_100) that had been predicted by RNA-seq data was confirmed, differing by the presence of an exon in the 5'-UTR (Supplementary Figure S7B). By analogy to the importance of AS in the 5'-UTR for regulating gene function in *A. thaliana* [2], our results indicate that such regulation may occur in *P. patens,* in addition to confirming the evolutionary conservation of SR gene splicing.

Pp1s28_193V6 (an SR34A homolog) belongs to the RS subfamily of SR proteins. Using RT-PCR, we identified two previously annotated isoforms of the Pp1s28_193V6 gene in protonemata, which differed by the retention of an intron at the 5'-UTR (Supplementary Figure S7C). Translation of the SR protein encoded by this gene was also confirmed in gametophores and protoplasts.

RNA-seq data revealed isoforms of the Pp1s332_29V6 (pp-RSZ23) gene, differing by the position of the second exon, and a higher expression level in protoplasts and gametophores compared to protonemata. Using RT-PCR, isoform Pp1s332_29V6.1 was identified, and a new isoform predicted by RNA-seq, which differed by intron retention in the second exon (Supplementary Figure S7D). We also discovered unique peptides, thus confirming the translation of these isoforms in gametophores and protonemata.

RT-PCR identified a single new isoform (TCONS_00067210) for the gene Pp1s9_312V6, which was predicted by RNA-seq; however, we failed to identify the predicted Pp1s9_312V6.1 isoform (Supplementary Figure S7E).

REFERENCES

1 Rauch, H. B. *et al.* Discovery and expression analysis of alternative splicing events conserved among plant SR proteins. *Molecular biology and evolution* **31**, 605-613, doi:10.1093/molbev/mst238 (2014).

2 Kalyna, M., Lopato, S. & Barta, A. Ectopic expression of atRSZ33 reveals its function in splicing and causes pleiotropic changes in development. *Molecular biology of the cell* **14**, 3565-3577, doi:10.1091/mbc.E03-02-0109 (2003).
